# Supplementary material for: Effectiveness of combining prevention psychological interventions with interventions that address the social determinants of mental health in low and middle-income countries: protocol of a systematic review and meta-analysis
Source: BMJ Open. 2024 May 16;14(5):e083261. doi: 10.1136/bmjopen-2023-083261 (PMC11103201; doi:10.1136/bmjopen-2023-083261)
Supplement: Supplementary data [file bmjopen-2023-083261supp001.pdf]

Effectiveness of combining prevention psychological interventions with interventions that address the social determinants of mental health in low- and middle- income countries: protocol of a systematic review and meta-analysis

Online supplementary material

Appendix 1

PRISMA-P (Preferred Reporting Items for Systematic review and Meta-Analysis Protocols) 2015 checklist: recommended items to address in a systematic review protocol*

Section and topic	Item No	Checklist item	Page
**ADMINISTRATIVE INFORMATION**			
Title:			
Identification	1a	Identify the report as a protocol of a systematic review	0
Update	1b	If the protocol is for an update of a previous systematic review, identify as such	NA
Registration	2	If registered, provide the name of the registry (such as PROSPERO) and registration number	1, 10
Authors:			
Contact	3a	Provide name, institutional affiliation, e-mail address of all protocol authors; provide physical mailing address of corresponding author	0
Contributions	3b	Describe contributions of protocol authors and identify the guarantor of the review	10
Amendments	4	If the protocol represents an amendment of a previously completed or published protocol, identify as such and list changes; otherwise, state plan for documenting important protocol amendments	NA
Support:			
Sources	5a	Indicate sources of financial or other support for the review	10
Sponsor	5b	Provide name for the review funder and/or sponsor	NA
Role of sponsor or funder	5c	Describe roles of funder(s), sponsor(s), and/or institution(s), if any, in developing the protocol	NA
**INTRODUCTION**			
Rationale	6	Describe the rationale for the review in the context of what is already known	3,4
Objectives	7	Provide an explicit statement of the question(s) the review will address with reference to participants, interventions, comparators, and outcomes (PICO)	4

**METHODS**			
Eligibility criteria	8	Specify the study characteristics (such as PICO, study design, setting, time frame) and report characteristics (such as years considered, language, publication status) to be used as criteria for eligibility for the review	4,5,6
Information sources	9	Describe all intended information sources (such as electronic databases, contact with study authors, trial registers or other grey literature sources) with planned dates of coverage	7
Search strategy	10	Present draft of search strategy to be used for at least one electronic database, including planned limits, such that it could be repeated	Appendix
Study records:			
Data management	11a	Describe the mechanism(s) that will be used to manage records and data throughout the review	7
Selection process	11b	State the process that will be used for selecting studies (such as two independent reviewers) through each phase of the review (that is, screening, eligibility and inclusion in meta-analysis)	7
Data collection process	11c	Describe planned method of extracting data from reports (such as piloting forms, done independently, in duplicate), any processes for obtaining and confirming data from investigators	7,8
Data items	12	List and define all variables for which data will be sought (such as PICO items, funding sources), any pre-planned data assumptions and simplifications	5,6,7,8
Outcomes and prioritization	13	List and define all outcomes for which data will be sought, including prioritization of main and additional outcomes, with rationale	6
Risk of bias in individual studies	14	Describe anticipated methods for assessing risk of bias of individual studies, including whether this will be done at the outcome or study level, or both; state how this information will be used in data synthesis	9
Data synthesis	15a	Describe criteria under which study data will be quantitatively synthesised	8
	15b	If data are appropriate for quantitative synthesis, describe planned summary measures, methods of handling data and methods of combining data from studies, including any planned exploration of consistency (such as I^2, Kendall's τ)	8,9
	15c	Describe any proposed additional analyses (such as sensitivity or subgroup analyses, meta-regression)	8,9
	15d	If quantitative synthesis is not appropriate, describe the type of summary planned	8
Meta-bias(es)	16	Specify any planned assessment of meta-bias(es) (such as publication bias across studies, selective reporting within studies)	8

Confidence in cumulative evidence	17	Describe how the strength of the body of evidence will be assessed (such as GRADE)	9

*** It is strongly recommended that this checklist be read in conjunction with the PRISMA-P Explanation and Elaboration (cite when available) for important clarification on the items. Amendments to a review protocol should be tracked and dated. The copyright for PRISMA-P (including checklist) is held by the PRISMA-P Group and is distributed under a Creative Commons Attribution Licence 4.0.**

From: Shamseer L, Moher D, Clarke M, Ghersi D, Liberati A, Petticrew M, Shekelle P, Stewart L, PRISMA-P Group. Preferred reporting items for systematic review and meta-analysis protocols (PRISMA-P) 2015: elaboration and explanation. BMJ. 2015 Jan 2;349(jan02 1):g7647.

Appendix 2

MEDLINE search

No	Search
1	psychosocial intervention/
2	exp mental disorders/
3	mania/
4	mental health/
5	depression/
6	child development/
7	mentally disabled persons/
8	exp self-injurious behavior/
9	(mental health* or mental* ill* or mental* disorder* or mental* well*).ti,ab,kf.
10	((substance or alcohol or opioid or morphine or marijuana or heroin or cocaine) adj2 (disorder* or illness* or dependence or abuse or misuse or "use")).ti,ab,kf.
11	(depressi* adj2 (sign* or symptom* or disorder*)).ti,ab,kf.
12	(depress* adj3 (acute or clinical* or diagnos* or disorder* or major or unipolar or illness or scale* or score* or adult* or child* or adolesc* or teen* or youth* or elder* or late* life* or patient* or participant* or people or inpatient* or in-patient* or outpatient* or out-patient*)).ti,ab,kf.
13	((depress* or distress*) adj3 (postnatal* or post natal* or maternal*)).ti,ab,kf.
14	(depression or anxiety or alzheimer* or schizoaffective or mania or manic or borderline personality or (stress adj2 disorder*) or adjustment disorder* or (psychological adj1 trauma*) or schizophrenia or psychoses or psychosis or stress syndrome* or distress syndrome* or combat disorder* or war disorder* or ptsd or dementia).ti,ab,kf.
15	((post-trauma* or posttrauma*) adj3 (stress* or disorder*)).ti,ab,kf.
16	(psychological trauma or psychotrauma*).ti,ab,kf.
17	(alcoholism or alcoholic* or drug addict* or drug abus* or drug misuse or drug user*).ti,ab,kf.
18	((learning or mental* or intellectual) adj (disabled or disabilit* or disorder* or difficult*)).ti,ab,kf.
19	((dissociative adj3 (disorder* or reaction*)) or dissociation).ti,ab,kf.

Prina E, *et al. BMJ Open* 2024; 14:e083261. doi: 10.1136/bmjopen-2023-083261

20	((bipolar or behavio?ral or obsessive or panic or mood or delusional) adj2 (disorder* or illness* or disease*)).ti,ab,kf.
21	(trichotillomani* or OCD or obsess*-compulsi* or GAD or stress reaction* or acute stress or neuros#s or neurotic).ti,ab,kf.
22	(affective* adj (disorder* or disease* or illness* or symptom*)).ti,ab,kf.
23	((mental or psychological or emotional or psycho-social or psychosocial) adj (stress* or distress*)).ti,ab,kf.
24	((sub-syndrom* or sub-threshold or sub-clinical or subsyndrom* or subthreshold or subclinical or minor or brief) adj (symptom* or disorder* or condition* or depress* or anxiety)).ti,ab,kf.
25	(mental relapse or fatigue or somatic symptom* or worry or worries or panic or low mood* or mood problem*).ti,ab,kf.
26	(anxiety disorder* or agoraphobi* or general* anxi* or separation anxiety or neurocirculatory asthenia or neurotic disorder* or social phobi* or self-harm* or self-injur* or suicid*).ti,ab,kf.
27	(slow* adj (thought* or think*)).ti,ab,kf.
28	(mental* adj develop*).ti,ab,kf.
29	or/2-28
30	primary health care/
31	physicians, family/
32	physicians, primary care/
33	general practitioners/
34	general practice/
35	family practice/
36	exp social support/
37	community health workers/
38	exp allied health personnel/
39	exp community health services/
40	schools/
41	school health services/ or school mental health services/
42	rural health/
43	rural population/
44	nurses, community health/
45	nurses, public health/
46	family nursing/

Prina E, *et al. BMJ Open* 2024; 14:e083261. doi: 10.1136/bmjopen-2023-083261

47	primary care nursing/
48	rural nursing/
49	community health nursing/
50	school nursing/
51	(primary adj5 (care or health*)).ti,ab,kf.
52	(family practi* or family doctor* or family physician* or gp* or general practi*).ti,ab,kf.
53	(school* or teacher* or rural* or community).ti,ab,kf.
54	(non-specialist* or nonspecialist* or social worker* or trainer*).ti,ab,kf.
55	(psycho-social or psychosocial).ti,ab,kf.
56	(caregiver* or care giver* or layperson*).ti,ab,kf.
57	(lay adj2 (heal* or person* or counsellor* or counselor* or worker* or therapist*)).ti,ab,kf.
58	(paraprofessional* or para-professional* or auxiliar* or paramedics or (allied health* adj (professional* or person* or staff or worker*)) or non-physician* or non-clinician*).ti,ab,kf.
59	(midwife or midwive* or pharmacist* or pharmacy or pharmacies or practice nurs* or district nurs* or health visitor*).ti,ab,kf.
60	(psychological intervention* or task shift* or shifting tasks or task sharing or sharing tasks or (delegate adj2 task*)).ti,ab,kf.
61	or/30-60
62	1 or (29 and 61)
63	(afghan* or africa* or albania* or algeria* or angola* or antigua* or barbuda* or argentin* or armenia* or aruba* or azerbaijan* or bahrain* or bangladesh* or bengal* or bangal* or barbados* or barbadian* or bajan or bajans or belarus* or belorus* or byelarus* or byelorus* or belize* or benin* or dahomey or bhutan* or bolivia* or bosnia* or herzegovin* or botswan* or batswan* or bechuanaland* or brazil* or brasil* or bulgaria* or burkina* or burkinese* or upper volta* or burundi* or urundi* or cabo verde* or cape verde* or cambodia* or kampuchea* or khmer* or cameroon* or cameroun* or ubangi shari* or chad* or chile* or china* or chinese or colombia* or comoro* or comore* or comorian* or mayotte* or congo* or zaire* or costa rica* or "cote d'ivoir*" or "cote d' ivoir*" or cote divoir* or cote d ivoir* or ivory coast* or ivorian* or croatia* or cuba or cuban or cubans or "cuba's" or cyprus* or cypriot* or czech* or djibouti* or french somaliland* or dominica* or ecuador* or egypt* or united arab republic* or el salvador* or salvadoran* or guinea* or equatoguinea* or eritrea* or estonia* or eswatini* or swaziland* or swazi* or swati* or ethiopia* or fiji* or gabon* or gabonese* or gabonaise* or gambia* or ((georgia or georgian or georgians) not (atlanta or california or florida)) or ghana* or gibraltar* or greece* or greek* or grecian* or grenada* or grenadian* or guam* or guatemala* or guyana* or guiana* or guyanese* or haiti* or hispaniola* or hondura* or hungary* or hungarian* or india* or indonesia* or iran* or iraq* or isle of man* or jamaica* or jordan* or kazakh* or

	kenya* or karabati* or korea* or kosovo* or kosova* or kyrgyz* or kirgiz* or kirghiz* or laos or lao or laotian* or latvia* or lebanon* or lebanese* or lesotho* or lesothan* or lesothonian* or basutoland* or mosotho* or basotho* or liberia* or libya* or jamahiriya* or lithuania* or macedonia* or madagasca* or malagasy* or malawi* or nyasaland* or malaysia* or malay* federation or maldives* or maldivian* or indian ocean or mali or malian* or "mali's" or malta or maltese* or "malta's" or micronesia* or marshallese* or kiribati* or marshall island* or nauru or nauran or nauruans or "naurian's" or mariana or marianas or palau or paluan* or tuvalu* or mauritania* or mauritan* or mauritius* or mexico* or mexican* or moldova* or moldovia* or mongol* or montenegr* or morocco* or moroccan* or ifni or mozambique* or mozambican* or myanmar* or burma* or burmese or namibia* or nepal* or new caledonia* or netherlands antill* or nicaragua* or niger* or oman or omani or omanis or "oman's" or pakistan* or palestin* or gaza* or west bank* or panama* or paraguay* or peru or peruvian* or "peru's" or philippine* or philipine* or phillipine* or phillippine* or filipino* or filipina* or poland* or polish or pole or poles or portugal* or portuguese or puerto ric* or romania* or russia* or ussr* or soviet* or rwanda* or rwandese or ruanda* or ruandese or samoa* or navigator island* or pacific island* or polynesia* or sao tome* or santomean* or saudi arabia* or saudi or saudis or senegal* or serbia* or seychell* or sierra leone* or slovak* or sloven* or melanesia* or solomon island* or norfolk island* or somali* or sri lanka* or ceylon* or saint kitts or st kitts or kittian* or nevisian* or saint lucia* or st lucia* or saint vincent* or st vincent* or vincentian* or grenadine* or sudan* or surinam* or syria* or tajik* or tadjik* or tadzhik* or tanzania* or tanganyika* or thai* or timor leste* or east timor* or timorese* or togo or togoles* or "togo's" or tonga* or trinidad* or tobago* or tunisia* or turkiy* or turkey* or turk or turks or turkish or turkmen* or uganda* or ukrain* or uruguay* or uzbek* or vanuatu* or new hebrides* or venezuela* or vietnam* or viet nam* or yemen* or yugoslav* or zambia* or zimbabwe* or rhodesia* or arab* countr* or middle east* or global south or sahara* or subsahara* or magreb* or maghrib* or west indies* or caribbean* or central america* or latin america* or south america* or asia central or central asia* or asia northern or north asia* or northern asia* or asia southeastern or southeastern asia* or south eastern asia* or southeast asia* or south east asia* or asia western or west asia* or western asia* or europe eastern or east europe* or eastern europe* or developing countr* or developing nation* or developing population* or developing world or less developed countr* or less developed nation* or less developed world or lesser developed countr* or lesser developed nation* or lesser developed world or under developed countr* or under developed nation* or under developed world or underdeveloped countr* or underdeveloped nation* or underdeveloped world or middle income countr* or middle income nation* or middle income population* or low income countr* or low income nation* or low income population* or lower income countr* or lower income nation* or lower income population* or underserved countr* or underserved nation* or underserved population* or under served population* or under served nation* or under served population* or deprived countr* or deprived population* or high burden countr* or high burden nation* or countdown countr* or countdown nation* or poor countr* or poor nation* or poor population* or poor world or poorer countr* or poorer nation* or poorer population* or poorer world or developing econom* or less developed econom* or underdeveloped econom* or under developed econom* or middle income econom* or low income econom* or lower income econom* or low gdp or low gnp or low

	gross domestic or low gross national or lower gdp or lower gnp or lower gross domestic or lower gross national or lmic or lmics or third world or lami countr* or transitional countr* or emerging econom* or emerging nation*).ti,ab,hw,kf.
64	exp randomized controlled trial/
65	controlled clinical trial.pt.
66	randomi#ed.ti,ab.
67	placebo.ab.
68	randomly.ti,ab.
69	Clinical Trials as topic.sh.
70	trial.ti.
71	or/64-70
72	exp animals/ not humans/
73	71 not 72 [Methods filter]
74	62 and 63 and 73
