# Supplementary material for: Effectiveness of combining prevention psychological interventions with interventions that address the social determinants of mental health in low and middle-income countries: protocol of a systematic review and meta-analysis
Source: BMJ Open. 2024 May 16;14(5):e083261. doi: 10.1136/bmjopen-2023-083261 (PMC11103201; doi:10.1136/bmjopen-2023-083261)
Supplement: Author's manuscript [file bmjopen-2023-083261.draft_revisions.pdf]

BMJ Open is committed to open peer review. As part of this commitment we make the peer review history of every article we publish publicly available.

When an article is published we post the peer reviewers' comments and the authors' responses online. We also post the versions of the paper that were used during peer review. These are the versions that the peer review comments apply to.

The versions of the paper that follow are the versions that were submitted during the peer review process. They are not the versions of record or the final published versions. They should not be cited or distributed as the published version of this manuscript.

BMJ Open is an open access journal and the full, final, typeset and author-corrected version of record of the manuscript is available on our site with no access controls, subscription charges or pay-per-view fees (http://bmjopen.bmj.com).

If you have any questions on BMJ Open's open peer review process please email info.bmjopen@bmj.com

BMJ Open

Effectiveness of combining prevention psychological interventions with interventions that address the social determinants of mental health in low- and middle- income countries: protocol of a systematic review and meta-analysis

Journal:	*BMJ Open*
Manuscript ID	bmjopen-2023-083261
Article Type:	Protocol
Date Submitted by the Author:	18-Dec-2023

Complete List of Authors:	Prina, Eleonora; University of Verona,<br>Bano, Beatrice; University of Verona, Department of Neuroscience, Biomedicine and Movement Sciences; Università della Svizzera italiana<br>Singh, Rakesh; Transcultural Psychosocial Organization Nepal<br>Albanese, Emiliano ; Università della Svizzera Italiana; University of Geneva, Department of Psychiatry<br>Trujillo, Daniela; Innovations for Poverty Action<br>Dedios Sanguineti, María Cecilia; Universidad de Los Andes, School of Government<br>Sorsdahl, Katherine; University of Cape Town, Alan J Flisher Centre for Public Mental Health, Department of Psychiatry and Mental Health<br>Luitel, Nagendra; Transcultural Psychosocial Organization (TPO) Nepal<br>Garman, Emily; University of Cape Town, Alan J Flisher Centre for Public Mental Health, Department of Psychiatry and Mental Health<br>purgato, marianna; Verona University, Neuroscience, Biomedicine and Movement Sciences; University of Verona, Cochrane Global Mental Health<br>Barbui, Corrado; University of Verona, Department of Neuroscience, Biomedicine and Movement Sciences; University of Verona, Cochrane Global Mental Health<br>Jordans, Mark J. D.; King's College London, Centre for Global Mental Health, Health Service and Population Research Department, Institute of Psychiatry, Psychology & Neuroscience<br>Lund, Crick; University of Cape Town, Alan J Flisher Centre for Public Mental Health, Department of Psychiatry and Mental Health; King's College London, Centre for Global Mental Health, Health Service and Population Research Department, Institute of Psychiatry, Psychology & Neuroscience

Keywords:	Primary Prevention, PSYCHIATRY, PUBLIC HEALTH

SCHOLARONE™
Manuscripts

Effectiveness of combining prevention psychological interventions with interventions that address the social determinants of mental health in low- and middle- income countries: protocol of a systematic review and meta-analysis

Eleonora Prina[1], Beatrice Bano[1,2], Rakesh Singh[3], Emiliano Albanese[2,4], Daniela Trujillo[5], Maria Cecilia Dedios Sanguineti[6], Katherine Sorsdahl[7], Nagendra Luitel[3], Emily Garman[7], Marianna Purgato[1,8], Corrado Barbui[1,8], Mark Jordans[9], Crick Lund[7,9]

[1] WHO Collaborating Centre for Research and Training in Mental Health and Service Evaluation, Department of Neuroscience, Biomedicine and Movement Sciences, Section of Psychiatry, University of Verona, Verona, Italy

[2] Faculty of Biomedical Sciences, Institute of Public Health, Università della Svizzera italiana, Lugano, Switzerland

[3] Transcultural Psychosocial Organization Nepal, Kathmandu, Nepal

[4] Department of Psychiatry, University of Geneva, Switzerland

[5] Innovations for Poverty Action, Colombia

[6] School of Government, Los Andes University, Bogotá, Colombia

[7] Alan J Flisher Centre for Public Mental Health, Department of Psychiatry and Mental Health, University of Cape Town, Cape Town, South Africa

[8] Cochrane Global Mental Health, University of Verona, Verona, Italy

[9] Centre for Global Mental Health, Health Service and Population Research Department, Institute of Psychiatry, Psychology & Neuroscience, King's College London, London, UK

Author for correspondence:

Eleonora Prina
WHO Collaborating Centre for Research and Training in Mental Health and Service Evaluation, Department of Neurosciences, Biomedicine and Movement Sciences, University of Verona, Italy
Email: eleonora.prina@univr.it

Word Count: 3694

For peer review only

Abstract

Introduction: Common mental health conditions (CMHCs), including depression, anxiety, and post-traumatic stress disorder (PTSD) are highly prevalent in low- and middle-income countries (LMICs). Preventive strategies combining psychological interventions with interventions addressing the social determinants of mental health may represent a key strategy for effectively preventing CMHCs. However, no systematic reviews have evaluated the effectiveness of these combined intervention strategies for preventing CMHC.

Methods and analysis: This systematic review will include randomized controlled trials (RCTs) focused on the effectiveness of interventions that combine preventive psychological interventions with interventions that address the social determinants of mental health in LMICs. Primary outcome is the frequency of depression, anxiety, or PTSD at post-intervention as determined by a formal diagnostic tool or any other standardized criteria. We will search Epistemonikos, Cochrane Controlled Trials Register (CENTRAL), MEDLINE, Embase, PsycINFO, CINAHL, Global Index Medicus, ClinicalTrials.gov (Ctgov), International Clinical Trials Registry Platform (ICTRP). Two reviewers will independently extract the data and evaluate the risk of bias of included studies using the Cochrane risk of bias tool2. Random-effects meta-analyses will be performed, and certainty of evidence will be rated using the Grading of Recommendations Assessment, Development and Evaluation (GRADE) approach.

Ethics and dissemination: This study uses data from published studies; therefore, ethical review is not required. Findings will be presented in a published manuscript.

Trial registration number: PROSPERO CRD42023451072

For peer review only

For peer review only - http://bmjopen.bmj.com/site/about/guidelines.xhtml

Strengths and limitations of this study

- This study aims to address an important scientific gap related to the lack of evidence-based interventions combining psychological and social ingredients for preventing common mental health conditions (CMHCs) in low- and middle- income countries (LMICs).
- The present review will be conducted following the Cochrane Handbook for Systematic Reviews of Interventions and the Preferred Reporting Items for Systematic reviews and Meta-analyses (PRISMA) 2020 guidelines for data reporting.
- We expect high degrees of clinical and methodological heterogeneity among the included studies, as they have been conducted across different types of settings (including humanitarian settings).

For peer review only

Introduction

Common mental health conditions (CMHCs), defined here as depression, anxiety, and post-traumatic stress disorder (PTSD), are highly prevalent in low- and middle-income countries (LMICs), and exert a significant contribution to the global burden of disease and disability [1–4]. The World Health Organization (WHO) estimates that the prevalence of CMHCs in conflict settings is at 22% [5], which is, conservatively, around five times higher than the general population.

In LMICs, a range of factors such as poverty, conflict exposure, malnutrition, gender-based violence, and social inequalities influence the prevalence, incidence, and prevention strategies of CMHCs [6–10]. Described as social determinants of mental health, these factors are the social and economic conditions that may have a direct influence on the prevalence and severity of mental disorders across the life course [11–13]. Evidence suggests that CMHC are strongly socially determined among populations worldwide [14].

The COVID-19 pandemic is an additional public health emergency that occurred against an already complex backdrop of psychological suffering [15–17]. The pandemic has not only exacerbated existing psychological challenges but has also significantly affected the social determinants of mental health. [18].

In LMICs, the gap between individuals in need of mental health interventions and those who actually receive such interventions is large, with a high burden associated with CMHCs [19]. WHO reports highlight persistent gaps in the availability of mental health resources, significant variation between high- and low- income countries and across regions, and the need for continued country-level investment in policies, plans, health services, and monitoring systems for mental health care [20]. The global mental health community, therefore, advocates for the implementation of prevention strategies as an important opportunity for addressing this mental health gap [21].

Prevention is an approach aimed at reducing the likelihood of future disorders in the general population or for people who are identified as being at risk of a disorder [22,23]. Psychological prevention interventions in LMICs include short, simple interventions, mostly delivered through a task-shifting approach defined by the WHO as *"the rational redistribution of tasks among health workforce teams"* [24]. Specific functions are shifted and reallocated, where appropriate, from highly-qualified health professionals (such as psychiatric, psychotherapists) to health workers with shorter training and fewer qualifications.

For peer review only

Preventive measures can range from universal, selective, and/or indicated interventions, according to the Institute of Medicine (IOM) Framework [22,25]. Psychological interventions are widely used in the prevention of CMHCs, as highlighted by the Mental Health Gap Action Program (mhGAP) Intervention Guide [26]. These interventions offer practical support, aim at enhancing coping strategies, hope, and resilience. Two recent Cochrane reviews focused on the effectiveness of prevention interventions. A systematic review of 113 randomized controlled trials (RCTs) with 32,992 participants across 39 LMICs identified a positive effect of prevention interventions on symptoms of depression (SMD -0.69, 95% CI -1.08 to -0.30), anxiety (MD -0.14, 95% CI -0.27 to -0.01), and PTSD (SMD -0.24, 95% CI -0.41 to -0.08) for adult populations [27]. Accordingly, a Cochrane review focused on prevention interventions in humanitarian settings, found that psychological counselling may be effective for reducing depressive symptoms (MD -7.50, 95% CI -9.19 to -5.81) and anxiety symptoms (MD -6.10, 95% CI –7.57 to -4.63) in adults [28]. However, none of the included RCTs provided data on the efficacy of interventions to prevent the onset of CMHCs (i.e., incidence) [29].

A recent Lancet Commission sought to align global mental health efforts with sustainable development goals (SDGs) and emphasized the importance of efforts to prevent mental health disorders [21]. While there is preliminary evidence supporting the effectiveness of psychological interventions in preventing mental health disorders, there is a notable absence of systematic reviews of trials combining psychological and social components.

This systematic review and meta-analysis aims to comprehensively evaluate the impact of interventions that include psychological ingredients and target the social determinants of mental health.

Method and analysis

This protocol is reported in line with Preferred Reporting Items for Systematic Reviews and Meta-Analysis (PRISMA-P) guidelines [30,31](Appendix 1).

Criteria for considering studies for this review

The inclusion criteria are reported below, and visually represented in Figure 1.

Type of studies

For peer review only - http://bmjopen.bmj.com/site/about/guidelines.xhtml

This systematic review and meta-analysis will include randomized controlled trials (RCTs). Both individual and cluster RCTs will be considered. We will include trials that employ a cross-over design, and we will use data from the first randomized stage only.

Type of participants and settings

We will include participants of any age, gender, ethnicity, and religion. Given the focus on prevention of CMHCs, we will exclude participants scoring above a validated cut-off on a symptom checklist at baseline or with a formal diagnosis of mental disorders at the time of recruitment, regardless of whether they have a physical health disability. We will focus on 1) the general population (universal prevention), 2) subpopulations exposed to risk factors, e.g. populations exposed to armed conflicts or natural disasters, those living in poverty or without social networks (selective prevention), and 3) individuals with increased psychological symptoms, but not meeting the criteria for a diagnosed mental disorder (indicated prevention).

We will include studies recruiting participants in any setting (e.g., healthcare setting, refugee camps, schools, home, community setting) in LMICs, as defined by the World Bank classification at the time of study conduction [32].

Type of interventions

We will include RCTs that combine psychological interventions with interventions that address the social determinants of mental health in LMICs. We will consider 1) interventions where both the social determinants and the psychological components are integrated into a single intervention (e.g., Strengthening Evidence base on scHool-based intErventions for pRomoting adolescent health (SEHER) programme conducted in India [33], that involve problem-solving counseling for health, social and academic issues, alongside school-wide strategies addressing themes like bullying, reproductive and sexual health, gender and violence, rights and responsibilities), 2) interventions in which the social and psychological components are delivered separately (e.g., in a trial in Tanzania [34], two programs were evaluated: the Skillful Program, a group-based intervention focusing on parenting skills and child protection, and the Agribusiness Training Program, which included three workshops covering access to credit for farm inputs, guidance on enhancing farming techniques, and establishing market connections).

The social determinants of mental health will be categorized into five primary domains, according to the theoretical framework of Lund et al [11]: demographic, economic, neighborhood, environmental events, and social/cultural. Within each domain, these determinants may exert influence on

For peer review only - http://bmjopen.bmj.com/site/about/guidelines.xhtml

individuals' mental health throughout their lives, involving a combination of distal and proximal factors. [11].

We will only include interventions delivered through task-shifting, e.g., by primary-level health workers (PHWs) and/or community workers (CWs) (i.e., interventions delivered by providers without a formal training in mental health and/or specialization with a redistribution or delegation of healthcare tasks within workforces and communities) [21,35,36]. Whenever possible, we will classify prevention interventions following the criteria of the Institute of Medicine [25] as universal, selective and indicated [22].

Interventions will be compared versus any control conditions such as no treatment, usual care, active control or waiting list.

Type of outcome measures

Our primary outcome is the frequency of depression, anxiety or post-traumatic stress disorder (PTSD) at post-intervention, as determined by a formal diagnostic tool (i.e., the Diagnostic and Statistical Manual of Mental Disorders (DSM) [37], International Classification of Diseases (ICD) [38] or any other standardized criteria, as assessed by scoring above a cut-off on a validated rating scale.

Our secondary outcomes are: the frequency of depression, anxiety or PTSD at one to six months post intervention, and at seven to 24 months post intervention; the symptoms of depression, anxiety and psychological distress at each timepoint; the changes in service utilization and contact coverage (including admission rates to hospital whether related to mental disorder or not; attendance rates with regards to utilization of primary or community services; or increased demand and/or referral rates from the primary/community setting to a mental health specialist); the resource use (for health services: personnel time allocated/ number of consultations, other opportunity costs of the intervention, or other aspects of the health service, assets; for participants: extra costs of travel, productivity, employment status, income, work absenteeism, retention, educational attainment, presenteeism).

We will group outcomes into three time points: a) post intervention (0 to 1 month after the intervention); b) follow-up 1 (1 to 6 months post intervention); c) follow-up 2 (7 to 24 months post intervention).

Search methods for identification of studies

We will adopt the search strategy of a recent Cochrane review of promotion and prevention interventions delivered through task-shifting approaches among children, adolescents and adults in LMICs [27]. In short, the following electronic databases were inspected without language restrictions:

For peer review only

Epistemonikos, Cochrane Controlled Trials Register (CENTRAL), MEDLINE (see appendix), Embase, PsycINFO, CINAHL, Global Index Medicus, ClinicalTrials.gov (Ctgov), International Clinical Trials Registry Platform (ICTRP). To ensure the robustness of the search strategy, we will cross check the results with those of a Cochrane review focused on mental health preventative strategies in humanitarian settings [28].

In addition to electronic search, we will perform a manual search of reference lists from relevant systematic reviews and primary studies related to this topic. We will document excluded and included studies following the Preferred Reporting Items for Systematic Review and Meta-analyses (PRISMA) guidelines [30].

Data collection and analysis

Selection of studies

All titles and abstracts identified through electronic searches will be downloaded into the reference management database (EndNote), and duplicates will be removed. The study selection process will be conducted independently by two reviewers (EP, BB, or RS) using the software Rayyan[39]. Any discrepancies will be resolved through consensus or, if necessary, by the decision of a third reviewer (CL, MP).

Data extraction and management

At least two reviewers (EP, BB, or RS) will extract descriptive characteristics and outcome data for the included studies using a standardized electronic spreadsheet. For all the included studies, the following information will be extracted: bibliographic information, setting, characteristics of participants, type of intervention (e.g. nature of the intervention, modality/method of delivery, domain of social determinants, type of prevention), type of comparator, outcome measure(s), effect estimates.

Prior to data collection, we will pilot the form on at least four included studies. Disagreements will be resolved by discussion or involving a third author (CL, MP). We will contact authors of included articles to request additional data not included in the original publication.

Data analysis

Measures of treatment effect

We will conduct meta-analyses using the software Cochrane Review Manager 5.4 [40]. As we expect some degree of heterogeneity, we will first apply a random-effects model. A fixed-effects model will

For peer review only - http://bmjopen.bmj.com/site/about/guidelines.xhtml

For peer review only

then be applied to check for any potential differences. We will estimate the effect of the intervention by using risk ratio (RR), together with the 95% confidence interval (CI), for primary and secondary (non-economic) outcomes. For continuous data, we will use mean differences when the same rating scale is applied to measure the outcome of interest. We will calculate standardized mean differences (SMD) wherever different scales were used in RCTs to measure the same outcome of interest, together with 95% confidence interval [31]. For economic data, we will present the results using a narrative approach.

If frequency of depression, anxiety or PTSD (primary outcome) are not available, we will first contact the authors of the studies. In the case of non-response, the frequency will be imputed according to commonly used cut-off scores for continuous measures of depressive, anxiety and PTSD symptoms from validated rating scales [41]. When standard deviations (SDs) are not reported and not supplied by authors on request, we will calculate them based on other measures reported in the study, for example, standard errors (SEs), t-statistics or P values, according to Altman [42]. For cluster-randomized controlled trails, we will include data adjusted with an intra-cluster correlation coefficient (ICC). If the ICC value is not reported or is not available from trial authors directly, we will assume it to be 0.05 [31].

If there is a sufficient number of included studies (n= 10), publication bias will be examined by inspecting the funnel plot.

Data synthesis

We will conduct separate meta-analysis for children and adolescents (≤ 19 years of age) and adults (>19 years of age). RCTs including mixed population groups will be allocated according to the proportion of participants belonging to the groups. In line with Cochrane methodology, we will not meta-analyze economic outcomes.

If there is a sufficient number of included studies, we will conduct the following subgroup analyses for the primary outcome: social determinants domain (demographic, economic, neighborhood, environmental events, and sociocultural), type of prevention (universal, selective, indicated), type of providers (PHWs, CWs), gender of the participants (male, female, others), country income at the time of study's implementation (low- or lower-middle- or upper-middle), humanitarian setting (vs non-humanitarian setting).

We will assess heterogeneity by visual inspection of forest plots, and using the I^2 statistics, following the interpretation suggested by the Cochrane handbook [43]: 0%–40%: might not be important; 30%–

For peer review only - http://bmjopen.bmj.com/site/about/guidelines.xhtml

60%: may represent moderate heterogeneity; 50%–90%: may represent substantial heterogeneity; 75%–100%: considerable heterogeneity.

We will perform sensitivity analyses for the primary outcome defined a priori to assess the robustness of our conclusions and to explore its impact on effect sizes. This will involve: excluding trials with a high risk of bias, excluding trials with methodological characteristics such as variability in study design (cluster RCT versus individual RCT) or outcome measurement tools that might generate the highest heterogeneity in meta-analyses (I^2> 75%).

Risk of bias and quality of included studies

Two independent reviewers (EP, BB) will apply the Cochrane risk-of-bias tool2 for RCTs (RoB 2.0 tool) [31]. This tool identifies key domains through which bias is likely to be included into trial design, conduct or analysis. For each of the five domains, the tool identifies potential source of bias, prompts the reviewer to provide support for a judgement, and requests the review author's judgement concerning the level of bias (low, some concerns, high risk of bias). We will apply the RoB 2.0 tool to the primary outcome. Any discrepancies will be resolved with a consensus between the two authors (EP, BB) and/or with a third reviewer (CL, MP). We will not exclude studies on the basis of their risk of bias. We will evaluate cluster-randomized controlled trials using specific section in the RoB 2.0 [31]. We will present our key findings in a "summary of findings' table using the Grading of Recommendations, Assessment, Development and Evaluation (GRADE) approach [43]. We will produce GRADE tables for the primary outcome.

Patient and public involvement

Patients and/or the public were not involved in the design, or conduct, or reporting, or dissemination plans of this research.

Ethics and dissemination

Ethical issues

The planned study relies on previously conducted studies and does not involve collection of new or identifiable data. Accordingly, no ethical review or informed consent is required.

Publication plan

This study has been registered in PROSPERO (https://www.crd.york.ac.uk/prospero/) (registration number: CRD42023451072). The authors plan to present findings of this review in a single published manuscript.

For peer review only - http://bmjopen.bmj.com/site/about/guidelines.xhtml

Competing interests statement

No potential conflict of interest was reported by the author(s).

Funding statement

RS, DT, MCD, KS, NL, EG, MJ and CL are supported by the National Institute for Health Research (NIHR) (using the UK's Official Development Assistance (ODA) Funding) and Wellcome (grant number: 221940/Z/20/Z) under the Department of Health and Social Care (DHSC)-Wellcome Partnership for Global Health Research. The views expressed are those of the authors and not necessarily those of the Wellcome Trust, NIHR or the DHSC.

For peer review only

References

[1] Global, regional, and national burden of 12 mental disorders in 204 countries and territories, 1990–2019: a systematic analysis for the Global Burden of Disease Study 2019. *Lancet Psychiatry* 2022;9:137–50. https://doi.org/10.1016/S2215-0366(21)00395-3.

[2] Li Y, Scherer N, Felix L, et al. Prevalence of depression, anxiety and post-traumatic stress disorder in health care workers during the COVID-19 pandemic: A systematic review and meta-analysis. *PLOS ONE* 2021;16:e0246454. https://doi.org/10.1371/journal.pone.0246454.

[3] Yatham S, Sivathasan S, Yoon R, et al. Depression, anxiety, and post-traumatic stress disorder among youth in low and middle income countries: A review of prevalence and treatment interventions. *Asian J Psychiatry* 2018;38:78–91. https://doi.org/10.1016/j.ajp.2017.10.029.

[4] Prina E, Ceccarelli C, Abdulmalik JO, et al. Task-sharing psychosocial interventions for the prevention of common mental disorders in the perinatal period in low- and middle-income countries: A systematic review and meta-analysis. *Int J Soc Psychiatry* 2023:207640231174451. https://doi.org/10.1177/00207640231174451.

[5] Charlson F, Van Ommeren M, Flaxman A, et al. New WHO prevalence estimates of mental disorders in conflict settings: a systematic review and meta-analysis. *The Lancet* 2019;394:240–8. https://doi.org/10.1016/S0140-6736(19)30934-1.

[6] Yasamy MT, Maulik PK, Tomlinson M, et al. Responsible Governance for Mental Health Research in Low Resource Countries. *PLoS Med* 2011;8:e1001126. https://doi.org/10.1371/journal.pmed.1001126.

[7] Kimutai JJ, Lund C, Moturi WN, et al. Evidence on the links between water insecurity, inadequate sanitation and mental health: A systematic review and meta-analysis. *PLoS ONE* 2023;18(5 May). https://doi.org/10.1371/journal.pone.0286146.

[8] Abrahams Z, Boisits S, Schneider M, et al. The relationship between common mental disorders (CMDs), food insecurity and domestic violence in pregnant women during the COVID-19 lockdown in Cape Town, South Africa. *Soc Psychiatry Psychiatr Epidemiol* 2022;57:37–46. https://doi.org/10.1007/s00127-021-02140-7.

[9] Lund C, Breen A, Flisher AJ, et al. Poverty and common mental disorders in low and middle income countries: A systematic review. *Soc Sci Med* 2010;71:517–28. https://doi.org/10.1016/j.socscimed.2010.04.027.

[10] Fisher J, Cabral de Mello M, Patel V, et al. Prevalence and determinants of common perinatal mental disorders in women in low- and lower-middle-income countries: a systematic review. *Bull World Health Organ* 2012;90:139-149H. https://doi.org/10.2471/BLT.11.091850.

For peer review only

[11] Lund C, Brooke-Sumner C, Baingana F, et al. Social determinants of mental disorders and the Sustainable Development Goals: a systematic review of reviews. *Lancet Psychiatry* 2018;5:357–69. https://doi.org/10.1016/S2215-0366(18)30060-9.

[12] Rose-Clarke K, Gurung D, Brooke-Sumner C, et al. Rethinking research on the social determinants of global mental health. *Lancet Psychiatry* 2020;7:659–62. https://doi.org/10.1016/S2215-0366(20)30134-6.

[13] Oswald T, Das-Munshi J, Hotopf M, et al. Evidence for public mental health interventions targeting the social determinants of mental disorders according to the Sustainable Development Goals: A systematic review of reviews. n.d.

[14] Lund C. Global mental health and its social determinants: How should we intervene? *Behav Res Ther* 2023;169:104402. https://doi.org/10.1016/j.brat.2023.104402.

[15] Santomauro DF, Mantilla Herrera AM, Shadid J, et al. Global prevalence and burden of depressive and anxiety disorders in 204 countries and territories in 2020 due to the COVID-19 pandemic. *The Lancet* 2021;398:1700–12. https://doi.org/10.1016/S0140-6736(21)02143-7.

[16] Prina E, Tedeschi F, Salazzari D, et al. Effect of COVID-19 pandemic on utilisation of community-based mental health care in North-East of Italy: A psychiatric case register study. *Epidemiol Psychiatr Sci* 2023;32:e17. https://doi.org/10.1017/S2045796023000100.

[17] Salanti G, Peter N, Tonia T, et al. The Impact of the COVID-19 Pandemic and Associated Control Measures on the Mental Health of the General Population: A Systematic Review and Dose–Response Meta-analysis. *Ann Intern Med* 2022;175:1560–71. https://doi.org/10.7326/M22-1507.

[18] Kola L, Kohrt BA, Hanlon C, et al. COVID-19 mental health impact and responses in low-income and middle-income countries: reimagining global mental health. *Lancet Psychiatry* 2021;8:535–50. https://doi.org/10.1016/S2215-0366(21)00025-0.

[19] Purgato M, Uphoff E, Singh R, et al. Promotion, prevention and treatment interventions for mental health in low- and middle-income countries through a task-shifting approach. *Epidemiol Psychiatr Sci* 2020;29:e150. https://doi.org/10.1017/S204579602000061X.

[20] World Health Organization. Mental Health Atlas 2020. 2021. www.who.int/publications/i/item/9789240036703 (accessed November 11, 2023).

[21] Patel V, Saxena S, Lund C, et al. The Lancet Commission on global mental health and sustainable development. *The Lancet* 2018;392:1553–98. https://doi.org/10.1016/S0140-6736(18)31612-X.

[22] Tol WA, Purgato M, Bass JK, et al. Mental health and psychosocial support in humanitarian settings: a public mental health perspective. *Epidemiol Psychiatr Sci* 2015;24:484–94. https://doi.org/10.1017/S2045796015000827.

For peer review only

[23] William W. Eaton. Public Mental Health. 2nd ed. New York, NY: Oxford University Press; 2019.

[24] World Health Organization Regional Office for Europe. Mental health promotion and mental health care in refugees and migrants. Technical guidance 2018. www.euro.who.int/__data/assets/pdf_file/0004/386563/ mental-health-eng.pdf.

[25] Institute of Medicine. Reducing Risks for Mental Disorders: Frontiers for Preventive Intervention Research. Washington, D.C.: *National Academies Press*; 1994. https://doi.org/10.17226/2139.

[26] World Health Organization. Mental Health Gap Action Programme (mhGAP). Geneva: World Health Organization; 2018.

[27] Purgato M, Prina E, Ceccarelli C, et al. Primary-level and community worker interventions for the prevention of mental disorders and the promotion of well-being in low- and middle-income countries. *Cochrane Database Syst Rev* 2023;2023. https://doi.org/10.1002/14651858.CD014722.pub2.

[28] Papola D, Purgato M, Gastaldon C, et al. Psychological and social interventions for the prevention of mental disorders in people living in low- and middle-income countries affected by humanitarian crises. *Cochrane Database Syst Rev* 2020;2020. https://doi.org/10.1002/14651858.CD012417.pub2.

[29] Cuijpers P. Why primary prevention often is no prevention at all. *Eur Neuropsychopharmacol* 2022;58:1–3. https://doi.org/10.1016/j.euroneuro.2022.01.004.

[30] Page MJ, McKenzie JE, Bossuyt PM, et al. The PRISMA 2020 statement: an updated guideline for reporting systematic reviews. *Syst Rev* 2021;10:89. https://doi.org/10.1186/s13643-021-01626-4.

[31] Julian H, James T, Jacqueline C, et al. Cochrane Handbook for Systematic Reviews of Interventions (6.3). 2021. https://training.cochrane.org/handbook.

[32] World Bank Country and Lending Groups. The World Bank 2022. https://datahelpdesk.worldbank.org/knowledgebase/articles/906519-world-bank-country-and-lending-groups.

[33] Shinde S, Weiss HA, Varghese B, et al. Promoting school climate and health outcomes with the SEHER multi-component secondary school intervention in Bihar, India: a cluster-randomised controlled trial. *The Lancet* 2018;392:2465–77. https://doi.org/10.1016/S0140-6736(18)31615-5.

[34] Lachman J, Wamoyi J, Spreckelsen T, et al. Combining parenting and economic strengthening programmes to reduce violence against children: a cluster randomised controlled trial with predominantly male caregivers in rural Tanzania. *BMJ Glob Health* 2020;5:e002349. https://doi.org/10.1136/bmjgh-2020-002349.

For peer review only

[35] WHO. Integrating mental health into primary care: a global perspective. 2018.

[36] Orkin AM, Rao S, Venugopal J, et al. Conceptual framework for task shifting and task sharing: an international Delphi study. *Hum Resour Health* 2021;19:61. https://doi.org/10.1186/s12960-021-00605-z.

[37] American Psychiatric Association. Diagnostic and Statistical Manual of Mental Disorders (DSM-5). 5th ed. 2013.

[38] World Health Organization. The Tenth Revision of the International Classification of Diseases and Related Health Problems (ICD-10). 1992.

[39] Ouzzani M, Hammady H, Fedorowicz Z, et al. Rayyan—a web and mobile app for systematic reviews. *Syst Rev* 2016;5:210. https://doi.org/10.1186/s13643-016-0384-4.

[40] Nordic Cochrane Centre, The Cochrane Collaboration Review Manager (RevMan). Review Manager 2020.

[41] Furukawa TA, Cipriani A, Barbui C, et al. Imputing response rates from means and standard deviations in meta-analyses: *Int Clin Psychopharmacol* 2005;20:49–52. https://doi.org/10.1097/00004850-200501000-00010.

[42] Altman DG, Bland JM. Statistics Notes: Detecting skewness from summary information. *BMJ* 1996;313:1200–1200. https://doi.org/10.1136/bmj.313.7066.1200.

[43] Guyatt GH, Oxman AD, Vist GE, et al. GRADE: an emerging consensus on rating quality of evidence and strength of recommendations. *BMJ* 2008;336:924–6. https://doi.org/10.1136/bmj.39489.470347.AD.

For peer review only

Figure 1. Causal pathway indicating inclusion criteria for the review.

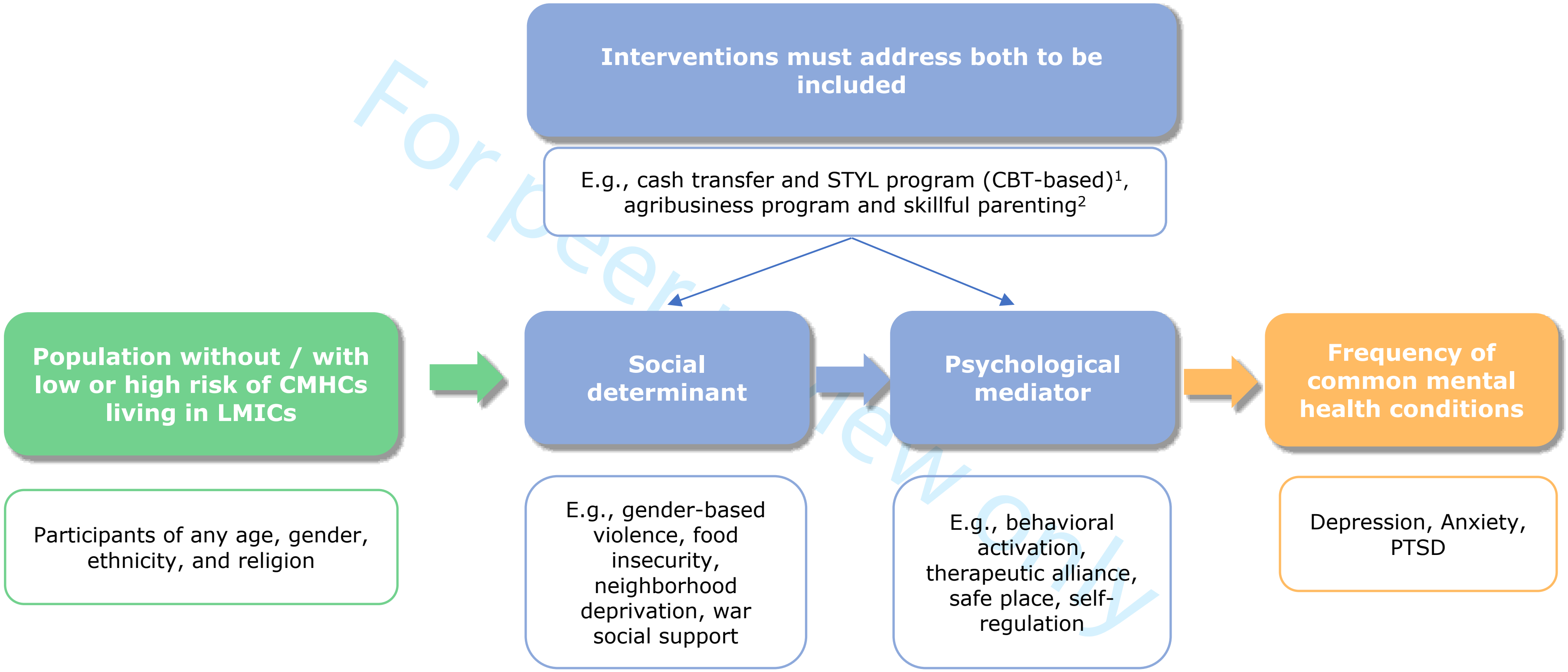

Notes

STYL: Sustainable transformation of youth in Liberia; CBT: Cognitive behavior therapy; CMHCs: Common mental health conditions; LMICs: Low- and middle- income countries; PTSD: Post traumatic stress disorder

[1] Blattman, Christopher, Julian C. Jamison, and Margaret Sheridan. 2017. "Reducing Crime and Violence: Experimental Evidence from Cognitive Behavioral Therapy in Liberia." American Economic Review, 107 (4): 1165-1206. doi: 10.1257/aer.20150503

[2] Lachman J, Wamoyi J, Spreckelsen T, et al. Combining parenting and economic strengthening programmes to reduce violence against children: a cluster randomised controlled trial with predominantly male caregivers in rural Tanzania. BMJ Global Health 2020;5:e002349. doi: 10.1136/ bmjgh-2020-002349

For peer review only

Effectiveness of combining prevention psychological interventions with interventions that address the social determinants of mental health in low- and middle- income countries: protocol of a systematic review and meta-analysis

Online supplementary material

For peer review only

Appendix 1

PRISMA-P (Preferred Reporting Items for Systematic review and Meta-Analysis Protocols) 2015 checklist: recommended items to address in a systematic review protocol*

Section and topic	Item No	Checklist item	Page
**ADMINISTRATIVE INFORMATION**			
Title:			
Identification	1a	Identify the report as a protocol of a systematic review	0
Update	1b	If the protocol is for an update of a previous systematic review, identify as such	NA
Registration	2	If registered, provide the name of the registry (such as PROSPERO) and registration number	1, 10
Authors:			
Contact	3a	Provide name, institutional affiliation, e-mail address of all protocol authors; provide physical mailing address of corresponding author	0
Contributions	3b	Describe contributions of protocol authors and identify the guarantor of the review	10
Amendments	4	If the protocol represents an amendment of a previously completed or published protocol, identify as such and list changes; otherwise, state plan for documenting important protocol amendments	NA
Support:			
Sources	5a	Indicate sources of financial or other support for the review	10
Sponsor	5b	Provide name for the review funder and/or sponsor	NA
Role of sponsor or funder	5c	Describe roles of funder(s), sponsor(s), and/or institution(s), if any, in developing the protocol	NA
**INTRODUCTION**			
Rationale	6	Describe the rationale for the review in the context of what is already known	3,4
Objectives	7	Provide an explicit statement of the question(s) the review will address with reference to participants, interventions, comparators, and outcomes (PICO)	4

For peer review only

**METHODS**			
Eligibility criteria	8	Specify the study characteristics (such as PICO, study design, setting, time frame) and report characteristics (such as years considered, language, publication status) to be used as criteria for eligibility for the review	4,5,6
Information sources	9	Describe all intended information sources (such as electronic databases, contact with study authors, trial registers or other grey literature sources) with planned dates of coverage	7
Search strategy	10	Present draft of search strategy to be used for at least one electronic database, including planned limits, such that it could be repeated	Appendix
Study records:			
Data management	11a	Describe the mechanism(s) that will be used to manage records and data throughout the review	7
Selection process	11b	State the process that will be used for selecting studies (such as two independent reviewers) through each phase of the review (that is, screening, eligibility and inclusion in meta-analysis)	7
Data collection process	11c	Describe planned method of extracting data from reports (such as piloting forms, done independently, in duplicate), any processes for obtaining and confirming data from investigators	7,8
Data items	12	List and define all variables for which data will be sought (such as PICO items, funding sources), any pre-planned data assumptions and simplifications	5,6,7,8
Outcomes and prioritization	13	List and define all outcomes for which data will be sought, including prioritization of main and additional outcomes, with rationale	6
Risk of bias in individual studies	14	Describe anticipated methods for assessing risk of bias of individual studies, including whether this will be done at the outcome or study level, or both; state how this information will be used in data synthesis	9
Data synthesis	15a	Describe criteria under which study data will be quantitatively synthesised	8
	15b	If data are appropriate for quantitative synthesis, describe planned summary measures, methods of handling data and methods of combining data from studies, including any planned exploration of consistency (such as I^2, Kendall's τ)	8,9
	15c	Describe any proposed additional analyses (such as sensitivity or subgroup analyses, meta-regression)	8,9
	15d	If quantitative synthesis is not appropriate, describe the type of summary planned	8
Meta-bias(es)	16	Specify any planned assessment of meta-bias(es) (such as publication bias across studies, selective reporting within studies)	8

For peer review only

Confidence in cumulative evidence	17	Describe how the strength of the body of evidence will be assessed (such as GRADE)	9

*** It is strongly recommended that this checklist be read in conjunction with the PRISMA-P Explanation and Elaboration (cite when available) for important clarification on the items. Amendments to a review protocol should be tracked and dated. The copyright for PRISMA-P (including checklist) is held by the PRISMA-P Group and is distributed under a Creative Commons Attribution Licence 4.0.**

From: Shamseer L, Moher D, Clarke M, Ghersi D, Liberati A, Petticrew M, Shekelle P, Stewart L, PRISMA-P Group. Preferred reporting items for systematic review and meta-analysis protocols (PRISMA-P) 2015: elaboration and explanation. BMJ. 2015 Jan 2;349(jan02 1):g7647.

For peer review only

Appendix 2

MEDLINE search

No	Search
1	psychosocial intervention/
2	exp mental disorders/
3	mania/
4	mental health/
5	depression/
6	child development/
7	mentally disabled persons/
8	exp self-injurious behavior/
9	(mental health* or mental* ill* or mental* disorder* or mental* well*).ti,ab,kf.
10	((substance or alcohol or opioid or morphine or marijuana or heroin or cocaine) adj2 (disorder* or illness* or dependence or abuse or misuse or "use")).ti,ab,kf.
11	(depressi* adj2 (sign* or symptom* or disorder*)).ti,ab,kf.
12	(depress* adj3 (acute or clinical* or diagnos* or disorder* or major or unipolar or illness or scale* or score* or adult* or child* or adolesc* or teen* or youth* or elder* or late* life* or patient* or participant* or people or inpatient* or in-patient* or outpatient* or out-patient*)).ti,ab,kf.
13	((depress* or distress*) adj3 (postnatal* or post natal* or maternal*)).ti,ab,kf.
14	(depression or anxiety or alzheimer* or schizoaffective or mania or manic or borderline personality or (stress adj2 disorder*) or adjustment disorder* or (psychological adj1 trauma*) or schizophrenia or psychoses or psychosis or stress syndrome* or distress syndrome* or combat disorder* or war disorder* or ptsd or dementia).ti,ab,kf.
15	((post-trauma* or posttrauma*) adj3 (stress* or disorder*)).ti,ab,kf.
16	(psychological trauma or psychotrauma*).ti,ab,kf.
17	(alcoholism or alcoholic* or drug addict* or drug abus* or drug misuse or drug user*).ti,ab,kf.
18	((learning or mental* or intellectual) adj (disabled or disabilit* or disorder* or difficult*)).ti,ab,kf.
19	((dissociative adj3 (disorder* or reaction*)) or dissociation).ti,ab,kf.

For peer review only

20	((bipolar or behavio?ral or obsessive or panic or mood or delusional) adj2 (disorder* or illness* or disease*)).ti,ab,kf.
21	(trichotillomani* or OCD or obsess*-compulsi* or GAD or stress reaction* or acute stress or neuros#s or neurotic).ti,ab,kf.
22	(affective* adj (disorder* or disease* or illness* or symptom*)).ti,ab,kf.
23	((mental or psychological or emotional or psycho-social or psychosocial) adj (stress* or distress*)).ti,ab,kf.
24	((sub-syndrom* or sub-threshold or sub-clinical or subsyndrom* or subthreshold or subclinical or minor or brief) adj (symptom* or disorder* or condition* or depress* or anxiety)).ti,ab,kf.
25	(mental relapse or fatigue or somatic symptom* or worry or worries or panic or low mood* or mood problem*).ti,ab,kf.
26	(anxiety disorder* or agoraphobi* or general* anxi* or separation anxiety or neurocirculatory asthenia or neurotic disorder* or social phobi* or self-harm* or self-injur* or suicid*).ti,ab,kf.
27	(slow* adj (thought* or think*)).ti,ab,kf.
28	(mental* adj develop*).ti,ab,kf.
29	or/2-28
30	primary health care/
31	physicians, family/
32	physicians, primary care/
33	general practitioners/
34	general practice/
35	family practice/
36	exp social support/
37	community health workers/
38	exp allied health personnel/
39	exp community health services/
40	schools/
41	school health services/ or school mental health services/
42	rural health/
43	rural population/
44	nurses, community health/
45	nurses, public health/
46	family nursing/

For peer review only

47	primary care nursing/
48	rural nursing/
49	community health nursing/
50	school nursing/
51	(primary adj5 (care or health*)).ti,ab,kf.
52	(family practi* or family doctor* or family physician* or gp* or general practi*).ti,ab,kf.
53	(school* or teacher* or rural* or community).ti,ab,kf.
54	(non-specialist* or nonspecialist* or social worker* or trainer*).ti,ab,kf.
55	(psycho-social or psychosocial).ti,ab,kf.
56	(caregiver* or care giver* or layperson*).ti,ab,kf.
57	(lay adj2 (heal* or person* or counsellor* or counselor* or worker* or therapist*)).ti,ab,kf.
58	(paraprofessional* or para-professional* or auxiliar* or paramedics or (allied health* adj (professional* or person* or staff or worker*)) or non-physician* or non-clinician*).ti,ab,kf.
59	(midwife or midwive* or pharmacist* or pharmacy or pharmacies or practice nurs* or district nurs* or health visitor*).ti,ab,kf.
60	(psychological intervention* or task shift* or shifting tasks or task sharing or sharing tasks or (delegate adj2 task*)).ti,ab,kf.
61	or/30-60
62	1 or (29 and 61)
63	(afghan* or africa* or albania* or algeria* or angola* or antigua* or barbuda* or argentin* or armenia* or aruba* or azerbaijan* or bahrain* or bangladesh* or bengal* or bangal* or barbados* or barbadian* or bajan or bajans or belarus* or belorus* or byelarus* or byelorus* or belize* or benin* or dahomey or bhutan* or bolivia* or bosnia* or herzegovin* or botswan* or batswan* or bechuanaland* or brazil* or brasil* or bulgaria* or burkina* or burkinese* or upper volta* or burundi* or urundi* or cabo verde* or cape verde* or cambodia* or kampuchea* or khmer* or cameroon* or cameroun* or ubangi shari* or chad* or chile* or china* or chinese or colombia* or comoro* or comore* or comorian* or mayotte* or congo* or zaire* or costa rica* or "cote d'ivoir*" or "cote d' ivoir*" or cote divoir* or cote d ivoir* or ivory coast* or ivorian* or croatia* or cuba or cuban or cubans or "cuba's" or cyprus* or cypriot* or czech* or djibouti* or french somaliland* or dominica* or ecuador* or egypt* or united arab republic* or el salvador* or salvadoran* or guinea* or equatoguinea* or eritrea* or estonia* or eswatini* or swaziland* or swazi* or swati* or ethiopia* or fiji* or gabon* or gabonese* or gabonaise* or gambia* or ((georgia or georgian or georgians) not (atlanta or california or florida)) or ghana* or gibraltar* or greece* or greek* or grecian* or grenada* or grenadian* or guam* or guatemala* or guyana* or guiana* or guyanese* or haiti* or hispaniola* or hondura* or hungary* or hungarian* or india* or indonesia* or iran* or iraq* or isle of man* or jamaica* or jordan* or kazakh* or

	kenya* or karabati* or korea* or kosovo* or kosova* or kyrgyz* or kirgiz* or kirghiz* or laos or lao or laotian* or latvia* or lebanon* or lebanese* or lesotho* or lesothan* or lesothonian* or basutoland* or mosotho* or basotho* or liberia* or libya* or jamahiriya* or lithuania* or macedonia* or madagasca* or malagasy* or malawi* or nyasaland* or malaysia* or malay* federation or maldives* or maldivian* or indian ocean or mali or malian* or "mali's" or malta or maltese* or "malta's" or micronesia* or marshallese* or kiribati* or marshall island* or nauru or nauran or nauruans or "naurian's" or mariana or marianas or palau or paluan* or tuvalu* or mauritania* or mauritan* or mauritius* or mexico* or mexican* or moldova* or moldovia* or mongol* or montenegr* or morocco* or moroccan* or ifni or mozambique* or mozambican* or myanmar* or burma* or burmese or namibia* or nepal* or new caledonia* or netherlands antill* or nicaragua* or niger* or oman or omani or omanis or "oman's" or pakistan* or palestin* or gaza* or west bank* or panama* or paraguay* or peru or peruvian* or "peru's" or philippine* or philipine* or phillipine* or phillippine* or filipino* or filipina* or poland* or polish or pole or poles or portugal* or portuguese or puerto ric* or romania* or russia* or ussr* or soviet* or rwanda* or rwandese or ruanda* or ruandese or samoa* or navigator island* or pacific island* or polynesia* or sao tome* or santomean* or saudi arabia* or saudi or saudis or senegal* or serbia* or seychell* or sierra leone* or slovak* or sloven* or melanesia* or solomon island* or norfolk island* or somali* or sri lanka* or ceylon* or saint kitts or st kitts or kittian* or nevisian* or saint lucia* or st lucia* or saint vincent* or st vincent* or vincentian* or grenadine* or sudan* or surinam* or syria* or tajik* or tadjik* or tadzhik* or tanzania* or tanganyika* or thai* or timor leste* or east timor* or timorese* or togo or togoles* or "togo's" or tonga* or trinidad* or tobago* or tunisia* or turkiy* or turkey* or turk or turks or turkish or turkmen* or uganda* or ukrain* or uruguay* or uzbek* or vanuatu* or new hebrides* or venezuela* or vietnam* or viet nam* or yemen* or yugoslav* or zambia* or zimbabwe* or rhodesia* or arab* countr* or middle east* or global south or sahara* or subsahara* or magreb* or maghrib* or west indies* or caribbean* or central america* or latin america* or south america* or asia central or central asia* or asia northern or north asia* or northern asia* or asia southeastern or southeastern asia* or south eastern asia* or southeast asia* or south east asia* or asia western or west asia* or western asia* or europe eastern or east europe* or eastern europe* or developing countr* or developing nation* or developing population* or developing world or less developed countr* or less developed nation* or less developed world or lesser developed countr* or lesser developed nation* or lesser developed world or under developed countr* or under developed nation* or under developed world or underdeveloped countr* or underdeveloped nation* or underdeveloped world or middle income countr* or middle income nation* or middle income population* or low income countr* or low income nation* or low income population* or lower income countr* or lower income nation* or lower income population* or underserved countr* or underserved nation* or underserved population* or under served population* or under served nation* or under served population* or deprived countr* or deprived population* or high burden countr* or high burden nation* or countdown countr* or countdown nation* or poor countr* or poor nation* or poor population* or poor world or poorer countr* or poorer nation* or poorer population* or poorer world or developing econom* or less developed econom* or underdeveloped econom* or under developed econom* or middle income econom* or low income econom* or lower income econom* or low gdp or low gnp or low

For peer review only - http://bmjopen.bmj.com/site/about/guidelines.xhtml

	gross domestic or low gross national or lower gdp or lower gnp or lower gross domestic or lower gross national or lmic or lmics or third world or lami countr* or transitional countr* or emerging econom* or emerging nation*).ti,ab,hw,kf.
64	exp randomized controlled trial/
65	controlled clinical trial.pt.
66	randomi#ed.ti,ab.
67	placebo.ab.
68	randomly.ti,ab.
69	Clinical Trials as topic.sh.
70	trial.ti.
71	or/64-70
72	exp animals/ not humans/
73	71 not 72 [Methods filter]
74	62 and 63 and 73

For peer review only

BMJ Open

Effectiveness of combining prevention psychological interventions with interventions that address the social determinants of mental health in low- and middle- income countries: protocol of a systematic review and meta-analysis

Journal:	*BMJ Open*
Manuscript ID	bmjopen-2023-083261.R1
Article Type:	Protocol
Date Submitted by the Author:	08-Apr-2024

Complete List of Authors:	Prina, Eleonora; University of Verona,<br>Bano, Beatrice; University of Verona, Department of Neuroscience, Biomedicine and Movement Sciences; Università della Svizzera italiana<br>Singh, Rakesh; Transcultural Psychosocial Organization Nepal<br>Albanese, Emiliano ; Università della Svizzera Italiana; University of Geneva, Department of Psychiatry<br>Trujillo, Daniela; Innovations for Poverty Action<br>Dedios Sanguineti, María Cecilia; Universidad de Los Andes, School of Government<br>Sorsdahl, Katherine; University of Cape Town, Alan J Flisher Centre for Public Mental Health, Department of Psychiatry and Mental Health<br>Luitel, Nagendra; Transcultural Psychosocial Organization (TPO) Nepal<br>Garman, Emily; University of Cape Town, Alan J Flisher Centre for Public Mental Health, Department of Psychiatry and Mental Health<br>purgato, marianna; Verona University, Neuroscience, Biomedicine and Movement Sciences; University of Verona, Cochrane Global Mental Health<br>Barbui, Corrado; University of Verona, Department of Neuroscience, Biomedicine and Movement Sciences; University of Verona, Cochrane Global Mental Health<br>Jordans, Mark J. D.; King's College London, Centre for Global Mental Health, Health Service and Population Research Department, Institute of Psychiatry, Psychology & Neuroscience<br>Lund, Crick; University of Cape Town, Alan J Flisher Centre for Public Mental Health, Department of Psychiatry and Mental Health; King's College London, Centre for Global Mental Health, Health Service and Population Research Department, Institute of Psychiatry, Psychology & Neuroscience

**Primary Subject Heading**:	Global health
Secondary Subject Heading:	Mental health, Global health, Public health
Keywords:	Primary Prevention, PSYCHIATRY, PUBLIC HEALTH

SCHOLARONE™
Manuscripts

Effectiveness of combining prevention psychological interventions with interventions that address the social determinants of mental health in low- and middle- income countries: protocol of a systematic review and meta-analysis

Eleonora Prina[1], Beatrice Bano[1,2], Rakesh Singh[3], Emiliano Albanese[2,4], Daniela Trujillo[5], Maria Cecilia Dedios Sanguineti[6], Katherine Sorsdahl[7], Nagendra Luitel[3], Emily Garman[7], Marianna Purgato[1,8], Corrado Barbui[1,8], Mark Jordans[9], Crick Lund[7,9]

[1] WHO Collaborating Centre for Research and Training in Mental Health and Service Evaluation, Department of Neuroscience, Biomedicine and Movement Sciences, Section of Psychiatry, University of Verona, Verona, Italy

[2] Faculty of Biomedical Sciences, Institute of Public Health, Università della Svizzera italiana, Lugano, Switzerland

[3] Transcultural Psychosocial Organization Nepal, Kathmandu, Nepal

[4] Department of Psychiatry, University of Geneva, Switzerland

[5] Innovations for Poverty Action, Colombia

[6] School of Government, Los Andes University, Bogotá, Colombia

[7] Alan J Flisher Centre for Public Mental Health, Department of Psychiatry and Mental Health, University of Cape Town, Cape Town, South Africa

[8] Cochrane Global Mental Health, University of Verona, Verona, Italy

[9] Centre for Global Mental Health, Health Service and Population Research Department, Institute of Psychiatry, Psychology & Neuroscience, King's College London, London, UK

Author for correspondence:

Eleonora Prina
WHO Collaborating Centre for Research and Training in Mental Health and Service Evaluation, Department of Neurosciences, Biomedicine and Movement Sciences, University of Verona, Italy
Email: eleonora.prina@univr.it

Word Count: 3694

For peer review only

Abstract

Introduction: Common mental health conditions (CMHCs), including depression, anxiety, and post-traumatic stress disorder (PTSD) are highly prevalent in low- and middle-income countries (LMICs). Preventive strategies combining psychological interventions with interventions addressing the social determinants of mental health may represent a key strategy for effectively preventing CMHCs. However, no systematic reviews have evaluated the effectiveness of these combined intervention strategies for preventing CMHC.

Methods and analysis: This systematic review will include randomized controlled trials (RCTs) focused on the effectiveness of interventions that combine preventive psychological interventions with interventions that address the social determinants of mental health in LMICs. Primary outcome is the frequency of depression, anxiety, or PTSD at post-intervention as determined by a formal diagnostic tool or any other standardized criteria. We will search Epistemonikos, Cochrane Controlled Trials Register (CENTRAL), MEDLINE, Embase, PsycINFO, CINAHL, Global Index Medicus, ClinicalTrials.gov (Ctgov), International Clinical Trials Registry Platform (ICTRP). Two reviewers will independently extract the data and evaluate the risk of bias of included studies using the Cochrane risk of bias tool2. Random-effects meta-analyses will be performed, and certainty of evidence will be rated using the Grading of Recommendations Assessment, Development and Evaluation (GRADE) approach.

Ethics and dissemination: This study uses data from published studies; therefore, ethical review is not required. Findings will be presented in a published manuscript.

Trial registration number: PROSPERO CRD42023451072

For peer review only

Strengths and limitations of this study

- This study aims to address an important scientific gap related to the lack of evidence-based interventions combining psychological and social ingredients for preventing common mental health conditions (CMHCs) in low- and middle- income countries (LMICs).
- The present review will be conducted following the Cochrane Handbook for Systematic Reviews of Interventions and the Preferred Reporting Items for Systematic reviews and Meta-analyses (PRISMA) 2020 guidelines for data reporting.
- We expect high degrees of clinical and methodological heterogeneity among the included studies, as they have been conducted across different types of settings (including humanitarian settings).

For peer review only

Introduction

Common mental health conditions (CMHCs), defined here as depression, anxiety, and post-traumatic stress disorder (PTSD), are highly prevalent in low- and middle-income countries (LMICs), and exert a significant contribution to the global burden of disease and disability [1–4]. The World Health Organization (WHO) estimates that the prevalence of CMHCs in conflict settings is at 22% [5], which is notably, around five times higher than the general population.

In LMICs, a range of factors such as poverty, conflict exposure, malnutrition, gender-based violence, and social inequalities influence the prevalence, incidence, and prevention strategies of CMHCs [6–10]. Described as social determinants of mental health, these factors are the social and economic conditions that may have a direct influence on the prevalence and severity of mental disorders across the life course [11–13]. Evidence suggests that CMHC are strongly socially determined among populations worldwide [14].

The COVID-19 pandemic has emerged as an additional public health crisis within an already complex setting of psychological distress [15–17]. The pandemic has not only exacerbated existing psychological challenges but has also significantly affected the social determinants of mental health. [18].

In LMICs, the gap between persons in need of mental health interventions and those accessing such services is large, with a high burden associated with CMHCs [19]. Reports from the WHO highlight persistent disparities in mental health resource availability, significant differences between high- and low- income countries, as well as across regions. They emphasize the need for sustained national-level investment in mental health care policies, plans, health services, and monitoring systems [20]. Therefore, the global mental health community advocates for the implementation of prevention strategies as an important opportunity for addressing this mental health gap [21].

Prevention is an approach aimed at reducing the likelihood of future disorders in the general population or for people who are identified as being at risk of a disorder [22,23]. Psychological prevention interventions in LMICs include brief, straightforward interventions, mostly delivered via a task-shifting approach defined by the WHO as *"the rational redistribution of tasks among health workforce teams"* [24]. Specific functions are shifted and reallocated, where appropriate, from highly-qualified health professionals (such as psychiatric, psychotherapists) to health workers with shorter training and fewer qualifications.

For peer review only

Preventive strategies can range from universal, selective, and/or indicated interventions according to the Institute of Medicine (IOM) Framework [22,25]. Specifically, universal prevention includes strategies for the whole population, independently from the exposure to risk factors. Selective prevention targets high risk subpopulations based on identifiable risk factors [26], and indicated prevention focuses on individuals with increased vulnerability for a disorder (i.e., increased psychological symptoms) but not meeting the criteria for a full-blown mental health condition [22, 25]. Psychological interventions are widely used in the prevention of CMHCs, as highlighted by the Mental Health Gap Action Program (mhGAP) Intervention Guide [27]. These interventions offer practical support, aimed at enhancing coping strategies, hope, and resilience. Two recent Cochrane reviews focused on the effectiveness of prevention interventions. A systematic review of 113 randomized controlled trials (RCTs) with 32,992 participants across 39 LMICs identified a positive effect of prevention interventions on symptoms of depression (SMD -0.69, 95% CI -1.08 to -0.30), anxiety (MD -0.14, 95% CI -0.27 to -0.01), and PTSD (SMD -0.24, 95% CI -0.41 to -0.08) for adult populations [28]. Accordingly, a Cochrane review focused on prevention interventions in humanitarian settings, found that psychological counselling may be effective for reducing depressive symptoms (MD -7.50, 95% CI -9.19 to -5.81) and anxiety symptoms (MD -6.10, 95% CI – 7.57 to -4.63) among individuals without a formal mental health diagnosis [29]. However, none of the included RCTs provided data on the efficacy of interventions to prevent the onset of CMHCs (i.e., incidence) [30].

A recent Lancet Commission sought to align global mental health efforts with sustainable development goals (SDGs) and emphasized the importance of efforts to prevent mental health disorders [21]. While there is preliminary evidence supporting the effectiveness of psychological interventions [28] and programs targeting the social determinants of mental health[31] in preventing mental health disorders, there is a notable absence of systematic reviews of trials combining social (i.e., seeking to change or improve a social determinant of mental health) and psychological components . Only through our review can we understand the efficacy of both ingredients (i.e., psychological, and social) and approach a 'component' meta-analysis.

This systematic review and meta-analysis aim to comprehensively evaluate the impact of interventions that include psychological ingredients and target the social determinants of mental health.

Method and analysis

For peer review only

For peer review only - http://bmjopen.bmj.com/site/about/guidelines.xhtml

The project started on December 1, 2023, and it is expected to be completed by July 31, 2024. This protocol is reported in line with Preferred Reporting Items for Systematic Reviews and Meta-Analysis (PRISMA-P) guidelines [32,33](Appendix 1).

Criteria for considering studies for this review

The inclusion criteria are reported below, and visually represented in Figure 1.

Type of studies

This systematic review and meta-analysis will include randomized controlled trials (RCTs). Both individual and cluster RCTs will be considered. We will include trials that employ a cross-over design, and we will use data from the first randomized stage only.

Type of participants and settings

We will include participants of any age, gender, ethnicity, and religion. Given the focus on prevention of CMHCs, we will exclude participants scoring above a validated cut-off on a symptom checklist at baseline or with a formal diagnosis of mental disorders at the time of recruitment, regardless of whether they have a physical health disability. We will focus on 1) the general population (universal prevention), 2) subpopulations exposed to risk factors, e.g. populations exposed to armed conflicts or natural disasters, those living in poverty or without social networks (selective prevention), and 3) individuals with increased psychological symptoms, but not meeting the criteria for a diagnosed mental disorder (indicated prevention).

We will include studies recruiting participants in any setting (e.g., healthcare setting, refugee camps, schools, home, community setting) in LMICs, as defined by the World Bank classification at the time of study conduction [34].

Type of interventions

We will include RCTs that combine psychological interventions with interventions that address the social determinants of mental health in LMICs. We will consider 1) interventions where both the social determinants and the psychological components are integrated into a single intervention (e.g., Strengthening Evidence base on scHool-based intErventions for pRomoting adolescent health (SEHER) programme conducted in India [35], that involve problem-solving counseling for health, social and academic issues, alongside school-wide strategies addressing themes like bullying, reproductive and sexual health, gender and violence, rights and responsibilities), 2) interventions in which the social and psychological components are delivered separately (e.g., in a trial in Tanzania [36], two programs

For peer review only

were evaluated: the Skillful Program, a group-based intervention focusing on parenting skills and child protection, and the Agribusiness Training Program, which included three workshops covering access to credit for farm inputs, guidance on enhancing farming techniques, and establishing market connections).

The social determinants of mental health will be categorized into five primary domains, according to the theoretical framework of Lund et al [11]: demographic, economic, neighborhood, environmental events, and social/cultural. Within each domain, these determinants may exert influence on individuals' mental health throughout their lives, involving a combination of distal and proximal factors. [11].

We will only include interventions delivered through task-shifting, e.g., by non-specialist health workers (PHWs) and/or community workers (CWs) (i.e., interventions delivered by providers without a formal training in mental health and/or specialization with a redistribution or delegation of healthcare tasks within workforces and communities) due to scalability and cost-effectiveness reasons [21,37,38]. Whenever possible, we will classify prevention interventions following the criteria of the Institute of Medicine [25] as universal, selective and indicated [22].

Interventions will be compared versus any control conditions such as no treatment, usual care, active control or waiting list.

Type of outcome measures

Our primary outcome is the frequency of depression, anxiety or post-traumatic stress disorder (PTSD) at post-intervention, as determined by a formal diagnostic tool (i.e., the Diagnostic and Statistical Manual of Mental Disorders (DSM) [39], International Classification of Diseases (ICD) [40]) or any other standardized criteria, as assessed by scoring above a cut-off on a validated rating scale.

Our secondary outcomes are: the frequency of depression, anxiety or PTSD at one to six months post intervention, and at seven to 24 months post intervention; the symptoms of depression, anxiety and psychological distress at each timepoint; the changes in service utilization and contact coverage (including admission rates to hospital whether related to mental disorder or not; attendance rates with regards to utilization of primary or community services; or increased demand and/or referral rates from the primary/community setting to a mental health specialist); the resource use (for health services: personnel time allocated/ number of consultations, other opportunity costs of the intervention, or other aspects of the health service, assets; for participants: extra costs of travel, productivity, employment status, income, work absenteeism, retention, educational attainment, presenteeism).

For peer review only - http://bmjopen.bmj.com/site/about/guidelines.xhtml

We will group outcomes into three time points: a) post intervention (0 to 1 month after the intervention); b) follow-up 1 (1 to 6 months post intervention); c) follow-up 2 (7 to 24 months post intervention).

Search methods for identification of studies

We will adopt the search strategy of a recent Cochrane review of promotion and prevention interventions delivered through task-shifting approaches among children, adolescents and adults in LMICs [28]. In short, the following electronic databases were inspected without language restrictions: Epistemonikos, Cochrane Controlled Trials Register (CENTRAL), MEDLINE (see appendix 2), Embase, PsycINFO, CINAHL, Global Index Medicus, ClinicalTrials.gov (Ctgov), International Clinical Trials Registry Platform (ICTRP). To ensure the robustness of the search strategy, we will cross check the results with those of a Cochrane review focused on mental health preventative strategies in humanitarian settings [29].

In addition to electronic search, we will perform a manual search of reference lists from relevant systematic reviews and primary studies related to this topic. We will document excluded and included studies following the Preferred Reporting Items for Systematic Review and Meta-analyses (PRISMA) guidelines [32].

Data collection and analysis

Selection of studies

All titles and abstracts identified through electronic searches will be downloaded into the reference management database (EndNote), and duplicates will be removed. The study selection process will be conducted independently by two reviewers (EP, BB, or RS) using the software Rayyan[41]. Any discrepancies will be resolved through consensus or, if necessary, by the decision of a third reviewer (CL, MP).

Data extraction and management

At least two reviewers (EP, BB, or RS) will extract descriptive characteristics and outcome data for the included studies using a standardized electronic spreadsheet. For all the included studies, the following information will be extracted: bibliographic information, setting, characteristics of participants, type of intervention (e.g. nature of the intervention, modality/method of delivery, domain of social determinants, type of prevention), type of comparator, outcome measure(s), effect estimates.

For peer review only - http://bmjopen.bmj.com/site/about/guidelines.xhtml

Prior to data collection, we will pilot the form on at least four included studies. Disagreements will be resolved by discussion or involving a third author (CL, MP). We will contact authors of included articles to request additional data not included in the original publication.

Data analysis

Measures of treatment effect

We will conduct meta-analyses using the software Cochrane Review Manager 5.4 [42]. As we expect some degree of heterogeneity, we will first apply a random-effects model. A fixed-effects model will then be applied to check for any potential differences. We will estimate the effect of the intervention by using risk ratio (RR), together with the 95% confidence interval (CI), for primary and secondary (non-economic) outcomes. For continuous data, we will use mean differences when the same rating scale is applied to measure the outcome of interest. We will calculate standardized mean differences (SMD) wherever different scales were used in RCTs to measure the same outcome of interest, together with 95% confidence interval [33]. For economic data, we will present the results using a narrative approach.

If frequency of depression, anxiety or PTSD (primary outcome) are not available, we will first contact the authors of the studies. In the case of non-response, the frequency will be imputed according to commonly used cut-off scores for continuous measures of depressive, anxiety and PTSD symptoms from validated rating scales [43]. When standard deviations (SDs) are not reported and not supplied by authors on request, we will calculate them based on other measures reported in the study, for example, standard errors (SEs), t-statistics or P values, according to Altman [44]. For cluster-randomized controlled trails, we will include data adjusted with an intra-cluster correlation coefficient (ICC). If the ICC value is not reported or is not available from trial authors directly, we will assume it to be 0.05 [33].

If there is a sufficient number of included studies (n= 10), publication bias will be examined by inspecting the funnel plot.

Data synthesis

We will conduct separate meta-analysis for children and adolescents (≤ 19 years of age) and adults (>19 years of age). RCTs including mixed population groups will be allocated according to the proportion of participants belonging to the groups. In line with Cochrane methodology, we will not meta-analyze economic outcomes.

For peer review only - http://bmjopen.bmj.com/site/about/guidelines.xhtml

If there is a sufficient number of included studies, we will conduct the following subgroup analyses for the primary outcome: social determinants domain (demographic, economic, neighborhood, environmental events, and sociocultural), type of prevention (universal, selective, indicated), type of providers (PHWs, CWs), gender of the participants (male, female, others), country income at the time of study's implementation (low- or lower-middle- or upper-middle), humanitarian setting (vs non-humanitarian setting). We will carefully assess the type of control groups and the effects as a function of the control group (and type of control group).

We will assess heterogeneity by visual inspection of forest plots, and using the I^2 statistics, following the interpretation suggested by the Cochrane handbook [43]: 0%–40%: might not be important; 30%–60%: may represent moderate heterogeneity; 50%–90%: may represent substantial heterogeneity; 75%–100%: considerable heterogeneity.

We will perform sensitivity analyses for the primary outcome defined a priori to assess the robustness of our conclusions and to explore its impact on effect sizes. This will involve: excluding trials with a high risk of bias, excluding trials with methodological characteristics such as variability in study design (cluster RCT versus individual RCT) or outcome measurement tools that might generate the highest heterogeneity in meta-analyses ($I^2 > 75\%$).

Risk of bias and quality of included studies

Two independent reviewers (EP, BB) will apply the Cochrane risk-of-bias tool2 for RCTs (RoB 2.0 tool) [33]. This tool identifies key domains through which bias is likely to be included into trial design, conduct or analysis. For each of the five domains, the tool identifies potential source of bias, prompts the reviewer to provide support for a judgement, and requests the review author's judgement concerning the level of bias (low, some concerns, high risk of bias). We will apply the RoB 2.0 tool to the primary outcome. Any discrepancies will be resolved with a consensus between the two authors (EP, BB) and/or with a third reviewer (CL, MP). We will not exclude studies on the basis of their risk of bias. We will evaluate cluster-randomized controlled trials using specific section in the RoB 2.0 [33]. We will present our key findings in a summary of findings' table using the Grading of Recommendations, Assessment, Development and Evaluation (GRADE) approach [45]. We will produce GRADE tables for the primary outcome.

For peer review only - http://bmjopen.bmj.com/site/about/guidelines.xhtml

Contributions

CL, MJ, CB, MP, EP made substantial contributions to the conception and design of the study. The first draft of the manuscript was written by EP and critically revised by all authors (EP, BB, RS, EA, DT, MCDS, KS, NL, EG, MP, CB, MJ, CL) regarding their content's expertise. All authors read and approved the final manuscript.

Patient and public involvement

Patients and/or the public were not involved in the design, or conduct, or reporting, or dissemination plans of this research.

Ethics and dissemination

Ethical issues

The planned study relies on previously conducted studies and does not involve collection of new or identifiable data. Accordingly, no ethical review or informed consent is required.

Publication plan

This study has been registered in PROSPERO (https://www.crd.york.ac.uk/prospero/) (registration number: CRD42023451072). The authors plan to present findings of this review in a single published manuscript.

Competing interests statement

No potential conflict of interest was reported by the author(s).

Funding statement

RS, DT, MCD, KS, NL, EG, MJ and CL are supported by the National Institute for Health Research (NIHR) (using the UK's Official Development Assistance (ODA) Funding) and Wellcome (grant number: 221940/Z/20/Z) under the Department of Health and Social Care (DHSC)-Wellcome Partnership for Global Health Research. The views expressed are those of the authors and not necessarily those of the Wellcome Trust, NIHR or the DHSC.

For peer review only

References

[1] Global, regional, and national burden of 12 mental disorders in 204 countries and territories, 1990–2019: a systematic analysis for the Global Burden of Disease Study 2019. Lancet Psychiatry 2022;9:137–50. https://doi.org/10.1016/S2215-0366(21)00395-3.

[2] Li Y, Scherer N, Felix L, Kuper H. Prevalence of depression, anxiety and post-traumatic stress disorder in health care workers during the COVID-19 pandemic: A systematic review and meta-analysis. PLOS ONE 2021;16:e0246454. https://doi.org/10.1371/journal.pone.0246454.

[3] Yatham S, Sivathasan S, Yoon R, Da Silva TL, Ravindran AV. Depression, anxiety, and post-traumatic stress disorder among youth in low and middle income countries: A review of prevalence and treatment interventions. Asian J Psychiatry 2018;38:78–91. https://doi.org/10.1016/j.ajp.2017.10.029.

[4] Prina E, Ceccarelli C, Abdulmalik JO, Amaddeo F, Cadorin C, Papola D, et al. Task-sharing psychosocial interventions for the prevention of common mental disorders in the perinatal period in low- and middle-income countries: A systematic review and meta-analysis. Int J Soc Psychiatry 2023:207640231174451. https://doi.org/10.1177/00207640231174451.

[5] Charlson F, Van Ommeren M, Flaxman A, Cornett J, Whiteford H, Saxena S. New WHO prevalence estimates of mental disorders in conflict settings: a systematic review and meta-analysis. The Lancet 2019;394:240–8. https://doi.org/10.1016/S0140-6736(19)30934-1.

[6] Yasamy MT, Maulik PK, Tomlinson M, Lund C, Van Ommeren M, Saxena S. Responsible Governance for Mental Health Research in Low Resource Countries. PLoS Med 2011;8:e1001126. https://doi.org/10.1371/journal.pmed.1001126.

[7] Kimutai JJ, Lund C, Moturi WN, Shewangizaw S, Feyasa M, Hanlon C. Evidence on the links between water insecurity, inadequate sanitation and mental health: A systematic review and meta-analysis. PLoS ONE Electron Resour 2023;18(5 May) (no pagination). https://doi.org/10.1371/journal.pone.0286146.

[8] Abrahams Z, Boisits S, Schneider M, Prince M, Lund C. The relationship between common mental disorders (CMDs), food insecurity and domestic violence in pregnant women during the COVID-19 lockdown in Cape Town, South Africa. Soc Psychiatry Psychiatr Epidemiol 2022;57:37–46. https://doi.org/10.1007/s00127-021-02140-7.

[9] Lund C, Breen A, Flisher AJ, Kakuma R, Corrigall J, Joska JA, et al. Poverty and common mental disorders in low and middle income countries: A systematic review. Soc Sci Med 2010;71:517–28. https://doi.org/10.1016/j.socscimed.2010.04.027.

[10] Fisher J, Cabral de Mello M, Patel V, Rahman A, Tran T, Holton S, et al. Prevalence and determinants of common perinatal mental disorders in women in low- and lower-middle-income countries: a systematic review. Bull World Health Organ 2012;90:139-149H. https://doi.org/10.2471/BLT.11.091850.

[11] Lund C, Brooke-Sumner C, Baingana F, Baron EC, Breuer E, Chandra P, et al. Social determinants of mental disorders and the Sustainable Development Goals: a systematic review of reviews. Lancet Psychiatry 2018;5:357–69. https://doi.org/10.1016/S2215-0366(18)30060-9.

[12] Rose-Clarke K, Gurung D, Brooke-Sumner C, Burgess R, Burns J, Kakuma R, et al. Rethinking research on the social determinants of global mental health. Lancet Psychiatry 2020;7:659–62. https://doi.org/10.1016/S2215-0366(20)30134-6.

[13] Tassia Oswald, Jayati Das-Munshi, Matthew Hotopf, Peter Schofield, Crick Lund, Minh Thu Nguyen, et al. Evidence for public mental health interventions targeting the social determinants of mental disorders according to the Sustainable Development Goals: A systematic review of reviews. n.d.

[14] Lund C. Global mental health and its social determinants: How should we intervene? Behav Res Ther 2023;169:104402. https://doi.org/10.1016/j.brat.2023.104402.

[15] Santomauro DF, Mantilla Herrera AM, Shadid J, Zheng P, Ashbaugh C, Pigott DM, et al. Global prevalence and burden of depressive and anxiety disorders in 204 countries and territories in

For peer review only

2020 due to the COVID-19 pandemic. The Lancet 2021;398:1700–12. https://doi.org/10.1016/S0140-6736(21)02143-7.

[16] Prina E, Tedeschi F, Salazzari D, Botte T, Ballarin M, Rabbi L, et al. Effect of COVID-19 pandemic on utilisation of community-based mental health care in North-East of Italy: A psychiatric case register study. Epidemiol Psychiatr Sci 2023;32:e17. https://doi.org/10.1017/S2045796023000100.

[17] Salanti G, Peter N, Tonia T, Holloway A, White IR, Darwish L, et al. The Impact of the COVID-19 Pandemic and Associated Control Measures on the Mental Health of the General Population: A Systematic Review and Dose–Response Meta-analysis. Ann Intern Med 2022;175:1560–71. https://doi.org/10.7326/M22-1507.

[18] Kola L, Kohrt BA, Hanlon C, Naslund JA, Sikander S, Balaji M, et al. COVID-19 mental health impact and responses in low-income and middle-income countries: reimagining global mental health. Lancet Psychiatry 2021;8:535–50. https://doi.org/10.1016/S2215-0366(21)00025-0.

[19] Purgato M, Uphoff E, Singh R, Thapa Pachya A, Abdulmalik J, van Ginneken N. Promotion, prevention and treatment interventions for mental health in low- and middle-income countries through a task-shifting approach. Epidemiol Psychiatr Sci 2020;29:e150. https://doi.org/10.1017/S204579602000061X.

[20] World Health Organization. Mental Health Atlas 2020. 2021. www.who.int/ publications/i/item/9789240036703 (accessed November 11, 2023).

[21] Patel V, Saxena S, Lund C, Thornicroft G, Baingana F, Bolton P, et al. The Lancet Commission on global mental health and sustainable development. The Lancet 2018;392:1553–98. https://doi.org/10.1016/S0140-6736(18)31612-X.

[22] Tol WA, Purgato M, Bass JK, Galappatti A, Eaton W. Mental health and psychosocial support in humanitarian settings: a public mental health perspective. Epidemiol Psychiatr Sci 2015;24:484–94. https://doi.org/10.1017/S2045796015000827.

[23] William W. Eaton. Public Mental Health. 2nd ed. New York, NY: Oxford University Press; 2019.

[24] World Health Organization Regional Office for Europe. Mental health promotion and mental health care in refugees and migrants. Technical guidance 2018. www.euro.who.int/__data/assets/pdf_file/0004/386563/ mental-health-eng.pdf.

[25] Institute of Medicine. Reducing Risks for Mental Disorders: Frontiers for Preventive Intervention Research. Washington, D.C.: National Academies Press; 1994. https://doi.org/10.17226/2139.

[26] Lund C, Jordans MJD, Garman E, Araya R, Avendano M, Bauer A, et al. Strengthening self-regulation and reducing poverty to prevent adolescent depression and anxiety: Rationale, approach and methods of the ALIVE interdisciplinary research collaboration in Colombia, Nepal and South Africa. Epidemiol Psychiatr Sci 2023;32:e69. https://doi.org/10.1017/S2045796023000811.

[27] World Health Organization. Mental Health Gap Action Programme (mhGAP). Geneva: World Health Organization; 2018.

[28] Purgato M, Prina E, Ceccarelli C, Cadorin C, Abdulmalik JO, Amaddeo F, et al. Primary-level and community worker interventions for the prevention of mental disorders and the promotion of well-being in low- and middle-income countries. Cochrane Database Syst Rev 2023;2023. https://doi.org/10.1002/14651858.CD014722.pub2.

[29] Papola D, Purgato M, Gastaldon C, Bovo C, van Ommeren M, Barbui C, et al. Psychological and social interventions for the prevention of mental disorders in people living in low- and middle-income countries affected by humanitarian crises. Cochrane Database Syst Rev 2020;2020. https://doi.org/10.1002/14651858.CD012417.pub2.

[30] Cuijpers P. Why primary prevention often is no prevention at all. Eur Neuropsychopharmacol 2022;58:1–3. https://doi.org/10.1016/j.euroneuro.2022.01.004.

[31] Oswald TK, Nguyen MT, Mirza L, Lund C, Jones HG, Crowley G, et al. Interventions targeting social determinants of mental disorders and the Sustainable Development Goals: a systematic review of reviews. Psychol Med 2024:1–25. https://doi.org/10.1017/S0033291724000333.

For peer review only

[32] Page MJ, McKenzie JE, Bossuyt PM, Boutron I, Hoffmann TC, Mulrow CD, et al. The PRISMA 2020 statement: an updated guideline for reporting systematic reviews. Syst Rev 2021;10:89. https://doi.org/10.1186/s13643-021-01626-4.

[33] Higgins Julian, Thomas James, Chandler Jacqueline, Cumpston Miranda, Li Tianjing, Page Matthew, et al. Cochrane Handbook for Systematic Reviews of Interventions (6.3). 2021. https://training.cochrane.org/handbook.

[34] World Bank Country and Lending Groups. The World Bank 2022. https://datahelpdesk.worldbank.org/knowledgebase/articles/906519-world-bank-country-and-lending-groups.

[35] Shinde S, Weiss HA, Varghese B, Khandeparkar P, Pereira B, Sharma A, et al. Promoting school climate and health outcomes with the SEHER multi-component secondary school intervention in Bihar, India: a cluster-randomised controlled trial. The Lancet 2018;392:2465–77. https://doi.org/10.1016/S0140-6736(18)31615-5.

[36] Lachman J, Wamoyi J, Spreckelsen T, Wight D, Maganga J, Gardner F. Combining parenting and economic strengthening programmes to reduce violence against children: a cluster randomised controlled trial with predominantly male caregivers in rural Tanzania. BMJ Glob Health 2020;5:e002349. https://doi.org/10.1136/bmjgh-2020-002349.

[37] WHO. Integrating mental health into primary care: a global perspective. 2018.

[38] Orkin AM, Rao S, Venugopal J, Kithulegoda N, Wegier P, Ritchie SD, et al. Conceptual framework for task shifting and task sharing: an international Delphi study. Hum Resour Health 2021;19:61. https://doi.org/10.1186/s12960-021-00605-z.

[39] American Psychiatric Association. Diagnostic and Statistical Manual of Mental Disorders (DSM-5). 5th ed. 2013.

[40] World Health Organization. The Tenth Revision of the International Classification of Diseases and Related Health Problems (ICD-10). 1992.

[41] Ouzzani M, Hammady H, Fedorowicz Z, Elmagarmid A. Rayyan—a web and mobile app for systematic reviews. Syst Rev 2016;5:210. https://doi.org/10.1186/s13643-016-0384-4.

[42] Nordic Cochrane Centre, The Cochrane Collaboration Review Manager (RevMan). Review Manager 2020.

[43] Furukawa TA, Cipriani A, Barbui C, Brambilla P, Watanabe N. Imputing response rates from means and standard deviations in meta-analyses: Int Clin Psychopharmacol 2005;20:49–52. https://doi.org/10.1097/00004850-200501000-00010.

[44] Altman DG, Bland JM. Statistics Notes: Detecting skewness from summary information. BMJ 1996;313:1200–1200. https://doi.org/10.1136/bmj.313.7066.1200.

[45] Guyatt GH, Oxman AD, Vist GE, Kunz R, Falck-Ytter Y, Alonso-Coello P, et al. GRADE: an emerging consensus on rating quality of evidence and strength of recommendations. BMJ 2008;336:924–6. https://doi.org/10.1136/bmj.39489.470347.AD.

For peer review only

Figure 1. Causal pathway indicating inclusion criteria for the review.

For peer review only

For peer review only - http://bmjopen.bmj.com/site/about/guidelines.xhtml

Figure 1. Causal pathway indicating inclusion criteria for the review.

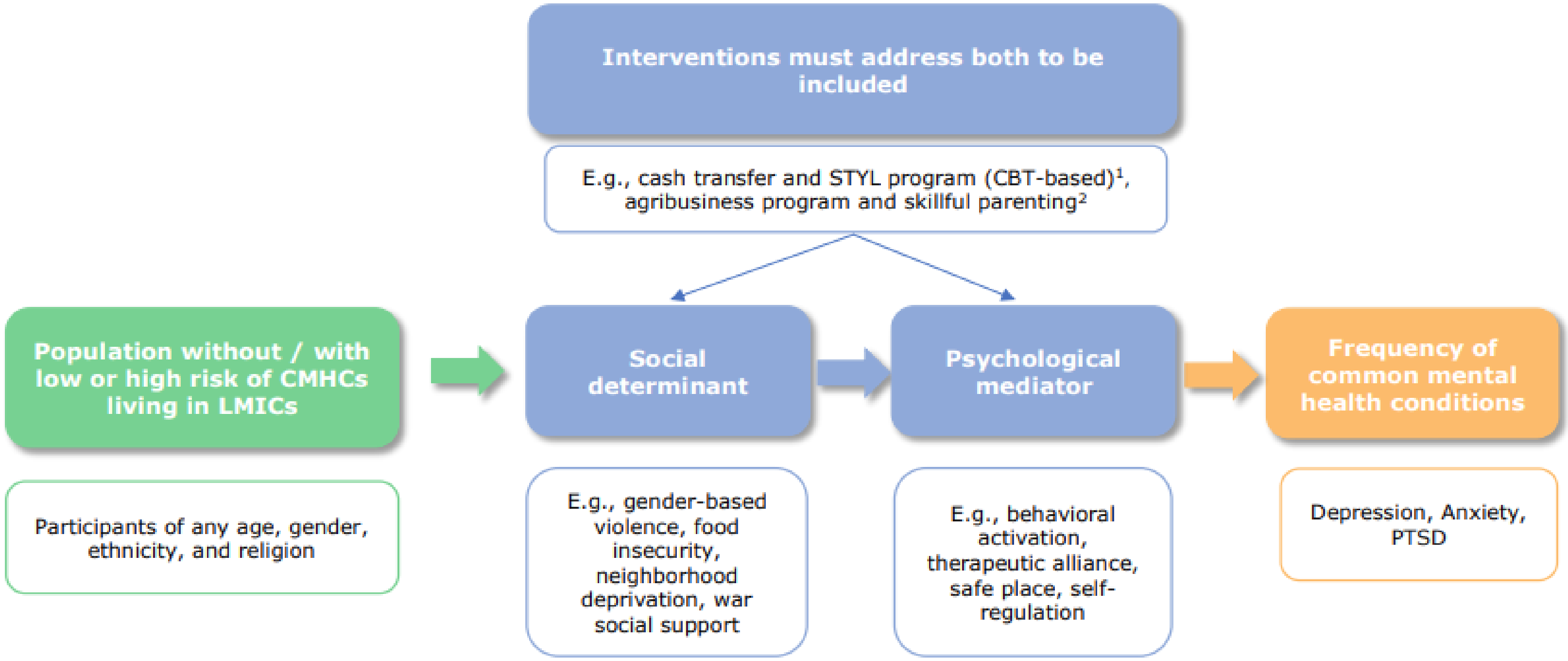

Notes

STYL: Sustainable transformation of youth in Liberia; CBT: Cognitive behavior therapy; CMHCs: Common mental health conditions; LMICs: Low- and middle- income countries; PTSD: Post traumatic stress disorder

[1] Blattman, Christopher, Julian C. Jamison, and Margaret Sheridan. 2017. "Reducing Crime and Violence: Experimental Evidence from Cognitive Behavioral Therapy in Liberia." American Economic Review, 107 (4): 1165-1206. doi: 10.1257/aer.20150503

[2] Lachman J, Wamoyi J, Spreckelsen T, et al. Combining parenting and economic strengthening programmes to reduce violence against children: a cluster randomised controlled trial with predominantly male caregivers in rural Tanzania. BMJ Global Health 2020;5:e002349. doi: 10.1136/ bmjgh-2020-002349

Effectiveness of combining prevention psychological interventions with interventions that address the social determinants of mental health in low- and middle- income countries: protocol of a systematic review and meta-analysis

For peer review only

Online supplementary material

Appendix 1

PRISMA-P (Preferred Reporting Items for Systematic review and Meta-Analysis Protocols) 2015 checklist: recommended items to address in a systematic review protocol*

Section and topic	Item No	Checklist item	Page
**ADMINISTRATIVE INFORMATION**			
Title:			
Identification	1a	Identify the report as a protocol of a systematic review	0
Update	1b	If the protocol is for an update of a previous systematic review, identify as such	NA
Registration	2	If registered, provide the name of the registry (such as PROSPERO) and registration number	1, 10
Authors:			
Contact	3a	Provide name, institutional affiliation, e-mail address of all protocol authors; provide physical mailing address of corresponding author	0
Contributions	3b	Describe contributions of protocol authors and identify the guarantor of the review	10
Amendments	4	If the protocol represents an amendment of a previously completed or published protocol, identify as such and list changes; otherwise, state plan for documenting important protocol amendments	NA
Support:			
Sources	5a	Indicate sources of financial or other support for the review	10
Sponsor	5b	Provide name for the review funder and/or sponsor	NA
Role of sponsor or funder	5c	Describe roles of funder(s), sponsor(s), and/or institution(s), if any, in developing the protocol	NA
**INTRODUCTION**			
Rationale	6	Describe the rationale for the review in the context of what is already known	3,4
Objectives	7	Provide an explicit statement of the question(s) the review will address with reference to participants, interventions, comparators, and outcomes (PICO)	4

For peer review only

**METHODS**			
Eligibility criteria	8	Specify the study characteristics (such as PICO, study design, setting, time frame) and report characteristics (such as years considered, language, publication status) to be used as criteria for eligibility for the review	4,5,6
Information sources	9	Describe all intended information sources (such as electronic databases, contact with study authors, trial registers or other grey literature sources) with planned dates of coverage	7
Search strategy	10	Present draft of search strategy to be used for at least one electronic database, including planned limits, such that it could be repeated	Appendix
Study records:			
Data management	11a	Describe the mechanism(s) that will be used to manage records and data throughout the review	7
Selection process	11b	State the process that will be used for selecting studies (such as two independent reviewers) through each phase of the review (that is, screening, eligibility and inclusion in meta-analysis)	7
Data collection process	11c	Describe planned method of extracting data from reports (such as piloting forms, done independently, in duplicate), any processes for obtaining and confirming data from investigators	7,8
Data items	12	List and define all variables for which data will be sought (such as PICO items, funding sources), any pre-planned data assumptions and simplifications	5,6,7,8
Outcomes and prioritization	13	List and define all outcomes for which data will be sought, including prioritization of main and additional outcomes, with rationale	6
Risk of bias in individual studies	14	Describe anticipated methods for assessing risk of bias of individual studies, including whether this will be done at the outcome or study level, or both; state how this information will be used in data synthesis	9
Data synthesis	15a	Describe criteria under which study data will be quantitatively synthesised	8
	15b	If data are appropriate for quantitative synthesis, describe planned summary measures, methods of handling data and methods of combining data from studies, including any planned exploration of consistency (such as I^2, Kendall's τ)	8,9
	15c	Describe any proposed additional analyses (such as sensitivity or subgroup analyses, meta-regression)	8,9
	15d	If quantitative synthesis is not appropriate, describe the type of summary planned	8
Meta-bias(es)	16	Specify any planned assessment of meta-bias(es) (such as publication bias across studies, selective reporting within studies)	8

For peer review only

Confidence in cumulative evidence	17	Describe how the strength of the body of evidence will be assessed (such as GRADE)	9

*** It is strongly recommended that this checklist be read in conjunction with the PRISMA-P Explanation and Elaboration (cite when available) for important clarification on the items. Amendments to a review protocol should be tracked and dated. The copyright for PRISMA-P (including checklist) is held by the PRISMA-P Group and is distributed under a Creative Commons Attribution Licence 4.0.**

From: Shamseer L, Moher D, Clarke M, Ghersi D, Liberati A, Petticrew M, Shekelle P, Stewart L, PRISMA-P Group. Preferred reporting items for systematic review and meta-analysis protocols (PRISMA-P) 2015: elaboration and explanation. BMJ. 2015 Jan 2;349(jan02 1):g7647.

For peer review only

Appendix 2

MEDLINE search

No	Search
1	psychosocial intervention/
2	exp mental disorders/
3	mania/
4	mental health/
5	depression/
6	child development/
7	mentally disabled persons/
8	exp self-injurious behavior/
9	(mental health* or mental* ill* or mental* disorder* or mental* well*).ti,ab,kf.
10	((substance or alcohol or opioid or morphine or marijuana or heroin or cocaine) adj2 (disorder* or illness* or dependence or abuse or misuse or "use")).ti,ab,kf.
11	(depressi* adj2 (sign* or symptom* or disorder*)).ti,ab,kf.
12	(depress* adj3 (acute or clinical* or diagnos* or disorder* or major or unipolar or illness or scale* or score* or adult* or child* or adolesc* or teen* or youth* or elder* or late* life* or patient* or participant* or people or inpatient* or in-patient* or outpatient* or out-patient*)).ti,ab,kf.
13	((depress* or distress*) adj3 (postnatal* or post natal* or maternal*)).ti,ab,kf.
14	(depression or anxiety or alzheimer* or schizoaffective or mania or manic or borderline personality or (stress adj2 disorder*) or adjustment disorder* or (psychological adj1 trauma*) or schizophrenia or psychoses or psychosis or stress syndrome* or distress syndrome* or combat disorder* or war disorder* or ptsd or dementia).ti,ab,kf.
15	((post-trauma* or posttrauma*) adj3 (stress* or disorder*)).ti,ab,kf.
16	(psychological trauma or psychotrauma*).ti,ab,kf.
17	(alcoholism or alcoholic* or drug addict* or drug abus* or drug misuse or drug user*).ti,ab,kf.
18	((learning or mental* or intellectual) adj (disabled or disabilit* or disorder* or difficult*)).ti,ab,kf.
19	((dissociative adj3 (disorder* or reaction*)) or dissociation).ti,ab,kf.

For peer review only

20	((bipolar or behavio?ral or obsessive or panic or mood or delusional) adj2 (disorder* or illness* or disease*)).ti,ab,kf.
21	(trichotillomani* or OCD or obsess*-compulsi* or GAD or stress reaction* or acute stress or neuros#s or neurotic).ti,ab,kf.
22	(affective* adj (disorder* or disease* or illness* or symptom*)).ti,ab,kf.
23	((mental or psychological or emotional or psycho-social or psychosocial) adj (stress* or distress*)).ti,ab,kf.
24	((sub-syndrom* or sub-threshold or sub-clinical or subsyndrom* or subthreshold or subclinical or minor or brief) adj (symptom* or disorder* or condition* or depress* or anxiety)).ti,ab,kf.
25	(mental relapse or fatigue or somatic symptom* or worry or worries or panic or low mood* or mood problem*).ti,ab,kf.
26	(anxiety disorder* or agoraphobi* or general* anxi* or separation anxiety or neurocirculatory asthenia or neurotic disorder* or social phobi* or self-harm* or self-injur* or suicid*).ti,ab,kf.
27	(slow* adj (thought* or think*)).ti,ab,kf.
28	(mental* adj develop*).ti,ab,kf.
29	or/2-28
30	primary health care/
31	physicians, family/
32	physicians, primary care/
33	general practitioners/
34	general practice/
35	family practice/
36	exp social support/
37	community health workers/
38	exp allied health personnel/
39	exp community health services/
40	schools/
41	school health services/ or school mental health services/
42	rural health/
43	rural population/
44	nurses, community health/
45	nurses, public health/
46	family nursing/

For peer review only

47	primary care nursing/
48	rural nursing/
49	community health nursing/
50	school nursing/
51	(primary adj5 (care or health*)).ti,ab,kf.
52	(family practi* or family doctor* or family physician* or gp* or general practi*).ti,ab,kf.
53	(school* or teacher* or rural* or community).ti,ab,kf.
54	(non-specialist* or nonspecialist* or social worker* or trainer*).ti,ab,kf.
55	(psycho-social or psychosocial).ti,ab,kf.
56	(caregiver* or care giver* or layperson*).ti,ab,kf.
57	(lay adj2 (heal* or person* or counsellor* or counselor* or worker* or therapist*)).ti,ab,kf.
58	(paraprofessional* or para-professional* or auxiliar* or paramedics or (allied health* adj (professional* or person* or staff or worker*)) or non-physician* or non-clinician*).ti,ab,kf.
59	(midwife or midwive* or pharmacist* or pharmacy or pharmacies or practice nurs* or district nurs* or health visitor*).ti,ab,kf.
60	(psychological intervention* or task shift* or shifting tasks or task sharing or sharing tasks or (delegate adj2 task*)).ti,ab,kf.
61	or/30-60
62	1 or (29 and 61)
63	(afghan* or africa* or albania* or algeria* or angola* or antigua* or barbuda* or argentin* or armenia* or aruba* or azerbaijan* or bahrain* or bangladesh* or bengal* or bangal* or barbados* or barbadian* or bajan or bajans or belarus* or belorus* or byelarus* or byelorus* or belize* or benin* or dahomey or bhutan* or bolivia* or bosnia* or herzegovin* or botswan* or batswan* or bechuanaland* or brazil* or brasil* or bulgaria* or burkina* or burkinese* or upper volta* or burundi* or urundi* or cabo verde* or cape verde* or cambodia* or kampuchea* or khmer* or cameroon* or cameroun* or ubangi shari* or chad* or chile* or china* or chinese or colombia* or comoro* or comore* or comorian* or mayotte* or congo* or zaire* or costa rica* or "cote d'ivoir*" or "cote d' ivoir*" or cote divoir* or cote d ivoir* or ivory coast* or ivorian* or croatia* or cuba or cuban or cubans or "cuba's" or cyprus* or cypriot* or czech* or djibouti* or french somaliland* or dominica* or ecuador* or egypt* or united arab republic* or el salvador* or salvadoran* or guinea* or equatoguinea* or eritrea* or estonia* or eswatini* or swaziland* or swazi* or swati* or ethiopia* or fiji* or gabon* or gabonese* or gabonaise* or gambia* or ((georgia or georgian or georgians) not (atlanta or california or florida)) or ghana* or gibraltar* or greece* or greek* or grecian* or grenada* or grenadian* or guam* or guatemala* or guyana* or guiana* or guyanese* or haiti* or hispaniola* or hondura* or hungary* or hungarian* or india* or indonesia* or iran* or iraq* or isle of man* or jamaica* or jordan* or kazakh* or

	kenya* or karabati* or korea* or kosovo* or kosova* or kyrgyz* or kirgiz* or kirghiz* or laos or lao or laotian* or latvia* or lebanon* or lebanese* or lesotho* or lesothan* or lesothonian* or basutoland* or mosotho* or basotho* or liberia* or libya* or jamahiriya* or lithuania* or macedonia* or madagasca* or malagasy* or malawi* or nyasaland* or malaysia* or malay* federation or maldives* or maldivian* or indian ocean or mali or malian* or "mali's" or malta or maltese* or "malta's" or micronesia* or marshallese* or kiribati* or marshall island* or nauru or nauran or nauruans or "naurian's" or mariana or marianas or palau or paluan* or tuvalu* or mauritania* or mauritan* or mauritius* or mexico* or mexican* or moldova* or moldovia* or mongol* or montenegr* or morocco* or moroccan* or ifni or mozambique* or mozambican* or myanmar* or burma* or burmese or namibia* or nepal* or new caledonia* or netherlands antill* or nicaragua* or niger* or oman or omani or omanis or "oman's" or pakistan* or palestin* or gaza* or west bank* or panama* or paraguay* or peru or peruvian* or "peru's" or philippine* or philipine* or phillipine* or phillippine* or filipino* or filipina* or poland* or polish or pole or poles or portugal* or portuguese or puerto ric* or romania* or russia* or ussr* or soviet* or rwanda* or rwandese or ruanda* or ruandese or samoa* or navigator island* or pacific island* or polynesia* or sao tome* or santomean* or saudi arabia* or saudi or saudis or senegal* or serbia* or seychell* or sierra leone* or slovak* or sloven* or melanesia* or solomon island* or norfolk island* or somali* or sri lanka* or ceylon* or saint kitts or st kitts or kittian* or nevisian* or saint lucia* or st lucia* or saint vincent* or st vincent* or vincentian* or grenadine* or sudan* or surinam* or syria* or tajik* or tadjik* or tadzhik* or tanzania* or tanganyika* or thai* or timor leste* or east timor* or timorese* or togo or togoles* or "togo's" or tonga* or trinidad* or tobago* or tunisia* or turkiy* or turkey* or turk or turks or turkish or turkmen* or uganda* or ukrain* or uruguay* or uzbek* or vanuatu* or new hebrides* or venezuela* or vietnam* or viet nam* or yemen* or yugoslav* or zambia* or zimbabwe* or rhodesia* or arab* countr* or middle east* or global south or sahara* or subsahara* or magreb* or maghrib* or west indies* or caribbean* or central america* or latin america* or south america* or asia central or central asia* or asia northern or north asia* or northern asia* or asia southeastern or southeastern asia* or south eastern asia* or southeast asia* or south east asia* or asia western or west asia* or western asia* or europe eastern or east europe* or eastern europe* or developing countr* or developing nation* or developing population* or developing world or less developed countr* or less developed nation* or less developed world or lesser developed countr* or lesser developed nation* or lesser developed world or under developed countr* or under developed nation* or under developed world or underdeveloped countr* or underdeveloped nation* or underdeveloped world or middle income countr* or middle income nation* or middle income population* or low income countr* or low income nation* or low income population* or lower income countr* or lower income nation* or lower income population* or underserved countr* or underserved nation* or underserved population* or under served population* or under served nation* or under served population* or deprived countr* or deprived population* or high burden countr* or high burden nation* or countdown countr* or countdown nation* or poor countr* or poor nation* or poor population* or poor world or poorer countr* or poorer nation* or poorer population* or poorer world or developing econom* or less developed econom* or underdeveloped econom* or under developed econom* or middle income econom* or low income econom* or lower income econom* or low gdp or low gnp or low

For peer review only

	gross domestic or low gross national or lower gdp or lower gnp or lower gross domestic or lower gross national or lmic or lmics or third world or lami countr* or transitional countr* or emerging econom* or emerging nation*).ti,ab,hw,kf.
64	exp randomized controlled trial/
65	controlled clinical trial.pt.
66	randomi#ed.ti,ab.
67	placebo.ab.
68	randomly.ti,ab.
69	Clinical Trials as topic.sh.
70	trial.ti.
71	or/64-70
72	exp animals/ not humans/
73	71 not 72 [Methods filter]
74	62 and 63 and 73

For peer review only
